# Characterizing the Discourse of Popular Diets to Describe Information Dispersal and Identify Leading Voices, Interaction, and Themes of Mental Health: Social Network Analysis

**DOI:** 10.2196/38245

**Published:** 2023-05-05

**Authors:** Melissa C Eaton, Yasmine C Probst, Marc A Smith

**Affiliations:** 1 School of Medical, Indigenous and Health Sciences University of Wollongong Wollongong Australia; 2 Social Media Research Foundation Redwood City, CA United States

**Keywords:** social media, popular diets, nutrition, public health, social network analysis

## Abstract

**Background:**

Social media has transformed the way health messages are communicated. This has created new challenges and ethical considerations while providing a platform to share nutrition information for communities to connect and for information to spread. However, research exploring the web-based diet communities of *popular diets* is limited.

**Objective:**

This study aims to characterize the web-based discourse of popular diets, describe information dissemination, identify influential voices, and explore interactions between community networks and themes of mental health.

**Methods:**

This exploratory study used Twitter social media posts for an online social network analysis. Popular diet keywords were systematically developed, and data were collected and analyzed using the NodeXL metrics tool (Social Media Research Foundation) to determine the key network metrics (vertices, edges, cluster algorithms, graph visualization, centrality measures, text analysis, and time-series analytics).

**Results:**

The vegan and ketogenic diets had the largest networks, whereas the zone diet had the smallest network. In total, 31.2% (54/173) of the top users endorsed the corresponding diet, and 11% (19/173) claimed a health or science education, which included 1.2% (2/173) of dietitians. Complete fragmentation and hub and spoke messaging were the dominant network structures. In total, 69% (11/16) of the networks interacted, where the ketogenic diet was mentioned most, with depression and anxiety and eating disorder words most prominent in the “zone diet” network and the least prominent in the “soy-free,” “vegan,” “dairy-free,” and “gluten-free” diet networks.

**Conclusions:**

Social media activity reflects diet trends and provides a platform for nutrition information to spread through resharing. A longitudinal exploration of popular diet networks is needed to further understand the impact social media can have on dietary choices. Social media training is vital, and nutrition professionals must work together as a community to actively reshare evidence-based posts on the web.

## Introduction

### Background

Social media, consisting of web-based networking sites such as Twitter, Facebook, and Instagram, has changed the way health messages are communicated [1], creating new challenges and ethical considerations [2]. In 2016, a total of 3.5 billion people worldwide were regular internet users, with more than two-thirds using social media [3]. In 2019, Facebook was the most popular global platform with 2.38 billion users, followed by YouTube, Instagram, TikTok, and Twitter [3]. The emergence of web-based social networking apps has allowed for novel research exploring social interactions, with data from the social networking site to be used [4]. Twitter, for example, enables the access to and analysis of big data over time, creating a means to study human behavior, interactions, and patterns through social network analysis (SNA) [4-6].

The SNA research methodology is primarily based on social network theory [5], suggesting that we understand social connectedness by analyzing the components of related experiences [7]. Over the last decade, SNA has been applied to health research related to physical activity, obesity, and policy change by way of understanding behavior and the transmission of information [8,9]. Social media has opened up the opportunity for SNA to be applied to web-based networks [8].

The web-based SNA methodology has been used in the disciplines of computer science, politics, climate change, education, and health [10-12]. In health, topic areas have included health-related conspiracy theories, public health messaging, and exploring the web-based discourse surrounding topical health and diseases such as COVID-19 [8,12,13]. However, to the best of our knowledge, there is no existing SNA in nutrition aimed at describing web-based discourse related to popular diets. Current research on social media and nutrition consists predominately of intervention, descriptive, and content analysis methods within target populations [14]. Although important, the growing influence and use of social media [3] and public nutrition messages [15] suggest that it is beneficial to consider the SNA methodology to allow for the exploration of patterns to monitor nutrition messages and to gain insights into how nutrition information is spread.

Credible public nutrition messages exist on the web [16]; however, misinformation is regularly shared, including potentially dangerous health messages [15,17,18] and pseudoscientific recommendations [19]. Furthermore, health and diet are 2 of the most common categories of misinformation on the web [18]. In particular, restrictive and popular dietary patterns, including fad diets [20,21], are forms of dietary misinformation that are regularly dispersed on the web [15]. Although there is evidence to support some popular diets in specific population groups [22,23], concern arises when restrictive diets are promoted to the general population using a one-size-fits-all approach.

The promotion of unbalanced nutrition information may be exaggerated on social media, with algorithms designed to show users similar content to what they interact with [24]. This may contribute to the development of an “echo chamber” [25], which can result in unbalanced views on a topic [24]. Kulshrestha et al [26] found this to be true for user intake of diet-related information on Twitter, with the diet content of interest heavily focused on only 1 or 2 topics [26]. As diet-related information consumed on the web can influence dietary choices [27,28], algorithms and “echo chambers” may enhance the promotion of popular diets [29]. This highlights the important role of dietitians and other qualified nutrition professionals on social media. It is vital for such professionals to be the trusted voice of nutrition in the web-based landscape, by sharing evidence-based health information, and to help identify and rectify dietary misinformation [30-32].

Furthermore, restrictive diets have been linked to negative physiological and psychological health outcomes, including eating disorders and depression and anxiety [21,33-35]. There is evidence to support restrictive dieting and body dissatisfaction as risk factors for depression [33,36,37], and anxiety has been associated with extreme dieting behaviors and binge eating [38-40]. These eating behaviors are also well-known risk factors for the development of both disordered eating and clinical eating disorders [41-43]. Therefore, it is important to explore how themes of mental health may exist within web-based diet networks.

Previous research has demonstrated the potential of social media to detect, identify, monitor, and classify mental health conditions [44-47]. De Choudhury et al [48] showed that social networking sites may be used to detect and identify populations with depression [48]. Karami et al [49] identified mental health as a common subtopic of diet-related conversations. In addition, Wilksche [50] found a negative association between social media and disordered eating behaviors in young adolescents following a content analysis of Twitter, whereas Zhou et al [44] found web-based eating disorder behavior described on Twitter to reflect that of offline eating disorder psychopathology. Therefore, as the web-based SNA methodology allows for the exploration of themes, text, and keywords within a network [51,52], this study aimed to explore the mental health themes within existing web-based popular diet networks.

### Objectives

This study provides novel insights into how nutrition information is dispersed, the key influential voices of popular diet networks on Twitter, describes how users interact, and explores any related themes of mental health. Owing to the exploratory nature, hypotheses will be generated from the outcomes of the analyses, adding insight for future research. The study objectives were to (1) explore network dissemination and how messages may spread, (2) identify key influential voices of each network, (3) explore the interaction between popular diet networks, and (4) explore the interaction between popular diet networks and mental health. Table 1 summarizes the objectives and their associated outcome measures.

**Table 1:** Summary of the research objectives and their associated outcome measures.

Research objectives	Associated outcome measures
Explore network dissemination and how messages may spread	Network size and duration of data Cluster algorithm and graph visualization
Identify key influential voices of each network	Betweenness centrality and out degree
Explore the interaction between popular diet networks	Text analysis of high-frequency hashtags
Explore the interaction between popular diet networks and mental health	Text analysis of mental health word list

## Methods

### Overview

This exploratory study used social media posts for an online SNA. Data were accessed from the social networking platform—Twitter, using the NodeXL SNA metrics tool [51]. NodeXL is a software plug-in for Microsoft Excel that allows the extraction, storage, analysis, and visualization of social network data [5,52,53]. NodeXL creates maps and visualizations of public conversations and connections between Twitter users [52]. Although ethically low risk as public data were extracted from Twitter, user names were deidentified by removing Twitter handles, and accounts were categorized using public profile data extracted via NodeXL. The categories included Twitter user (account identity), web-based business (business type), video-sharing platform, government initiative, community initiative, nonprofit initiative, television personality or host (related show or identity), actor, brand (brand type), web-based marketing company, personal brand (brand identity), politician, science- or health-related occupation (eg, Dr, Medical Doctor, PhD, and dietitian), band (band name), and unknown.

### Keyword Development

A systematic search strategy was used to source tweets (posts) from Twitter. The strategy was developed from a review by Ge et al [54] and Obert et al [55] to devise a list of keywords representing popular diets. To allow for potential colloquialisms associated with diets when discussed on the web, keywords derived from the coding used by Ramachandran et al [15] were also included.

To identify popular diets that were more restrictive in nature, the diet list was assessed by 2 independent reviewers (ME and YP) against a published definition. Diets were included if they promoted promises of weight loss [21] and either (1) suggested intake of macronutrients in particular proportions or (2) avoidance of particular foods or macronutrients [20].

The resulting popular diets formed the initial keywords that underwent 2 stages of feasibility testing using NodeXL. First, each keyword was individually searched at a rate limit of 1000 tweets. The keywords were excluded if the search (1) identified “no users in the network” (which would result in no edges) or (2) produced limited results consisting of <5 unique edges, which signaled a unique connection or interaction between 2 vertices (users).

**Figure 1:**
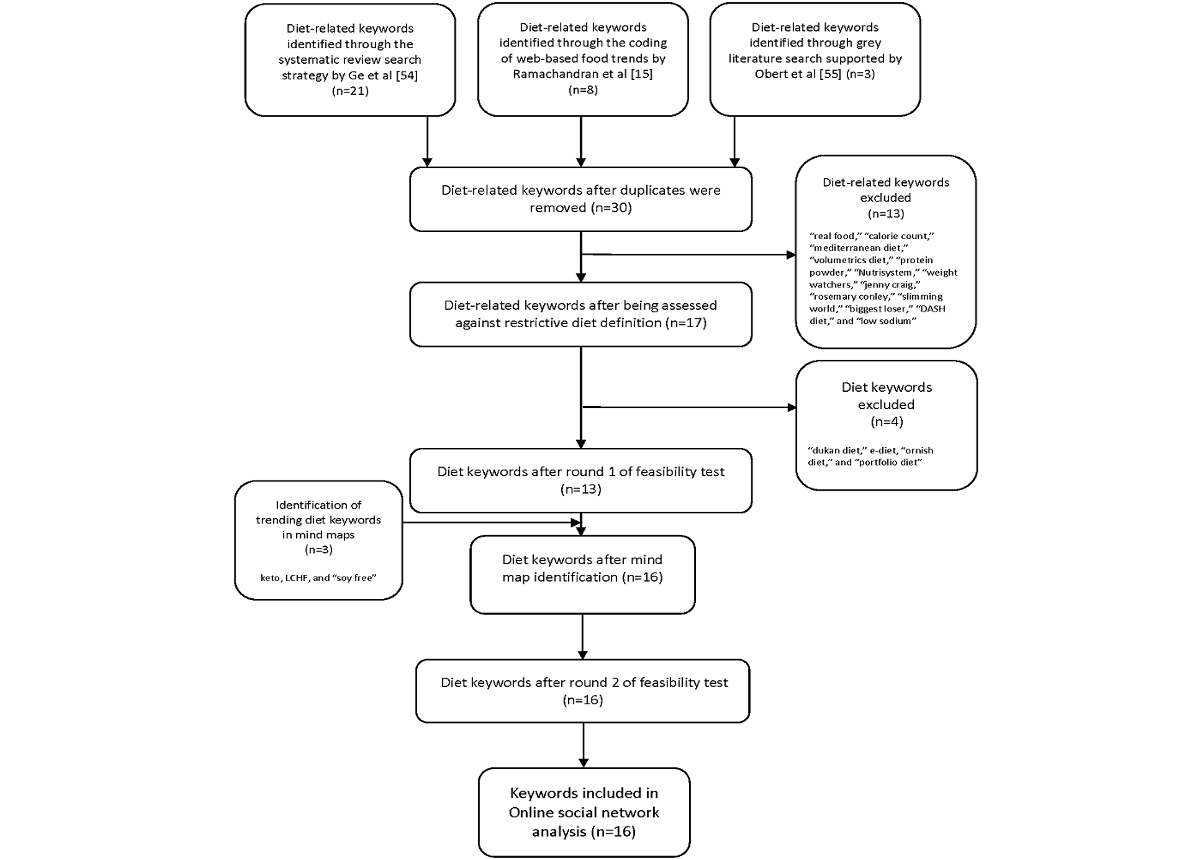
Flow diagram of keyword development.

To ensure that current and trending web-based popular diet discussions were captured and to identify gaps that were not previously captured in the literature, the top 10 hashtags of all the included keywords were identified using a network map (Multimedia Appendix 1 [52,56]; Figure 1). Hashtags were selected over “top words” because of their ability to categorize and pinpoint key messages on a topic [57-59]. Hashtags that were unrelated (eg, #catsoftwitter and #dinosaur) or that were more generally related to diet and health (eg, #diet, #weightloss, #health, #recipe, #healthy, and #food) were excluded. New keywords were included if they (1) fulfilled the inclusion criteria listed above in *Keyword Development* and (2) had at least 2 connections to the previously established keywords.

The top hashtags were also used to determine the validity of words that could represent popular diets chosen for ethical or medical reasons, such as vegan, “gluten free,” and “dairy free.” These keywords were considered appropriate for inclusion if they had at least 1 connection in the network map with the previously established keywords (Multimedia Appendix 1; Figure 1).

Second, individual keywords were searched in NodeXL at a rate limit of 5000 tweets to assess the web-based network for specificity and relevance. The keyword strategy outlined above in *Keyword Development* was further refined if the extracted data contained polysemy words, resulting in irrelevant topics (ie, words that had multiple meanings and were therefore not relevant). Two reviewers (ME and MS) independently reviewed the updated keyword strategy. A flow diagram outlining the keyword development process is shown in Figure 1.

### Data Extraction

After verifying the keywords, data from Twitter were extracted using NodeXL at a predetermined rate limit of 20,000 tweets [51] to ensure that the networks were comparable. The keywords selected for data collection included the following: paleo (food OR diet OR meal OR dine OR eat OR keto OR “low carb” OR “gluten free” OR “weight loss” OR “healthy” OR “recipes”); “raw food”; vegan; “sugar free”; “dairy free”; “gluten free”; “low carb”; “low fat”; “zone diet”; “atkins diet”; “south beach diet”; “keto”; “intermittent fasting”; “detox diet”; “LCHF”; and “soy free.” Each keyword was searched individually using NodeXL, and each network included all data that were publicly available at the time of extraction. This included Twitter handles, usernames, user bios, user tweets, user mentions and reshares, URLs, hashtags within tweets, user profile images, tweet date and time, and user country.

Data were collected daily in July 2020 from tweets spanning May 12 to July 20, 2020. The timeframe was dictated by the availability of tweets in each network (ie, with some spanning longer or shorter periods depending on the network size). Of note, the smaller networks spanned over a longer period, with more time needed to reach the predetermined threshold of 20,000. In some cases, data collection did not reach the threshold. The threshold was selected because it allowed for processing on available desktop and laptop computing resources and created multiweek and overlapping data sets [60].

The public Twitter application programming interface (API) was used because access to other APIs was limited at the time of extraction. It should be noted that although this API has limitations, it does not contain false positives, and limitations are present only in the form of false negatives [61,62]. To address this issue, repeated daily data collection was performed.

### Data Analysis

Data were analyzed using NodeXL through the application of network metrics [53]. All network metrics serve the function of analyzing connections and patterns that exist within the data set [52]. The specific metrics include vertices, edges, cluster algorithms, graph visualization, text analysis, centrality measures (in-degree, out degree, and betweenness), and time-series analytics. For definitions of the network metrics, refer to Multimedia Appendix 1.

### Network Message Dissemination

Vertices and time-series analytics were used to determine the size of the individual networks. Vertices or vertex, otherwise known as social media handles, correspond to the number of users within a network, whereas time-series metrics identify the time frame of data collection. A scatter plot was created to determine the size of the network across both dimensions.

Cluster algorithms and graph visualization were used to analyze the social connections between users [53]. An edge, also known as a relationship, tie, or link, represents the connection or interaction between 2 vertices (users)—visualized as a line between users [53]. Graph metrics visualize interactions, and from this, structures emerge by forming patterns and relationships that can be analyzed [53]. Social media form a range of network structures that reflect the social process that generates them. The divided, unified, fragmented, clustered, and in-hub and out-hub patterns each capture a common structure found on social media platforms such as Twitter [63]. Refer to Multimedia Appendix 1 for figure structure visualizations. Cluster algorithms identify, group, and analyze network vertices (users) that have shared characteristics [51]. The Clauset-Newman-Moore cluster algorithm, designed to extract community structures from networks [64], was used to group the network vertices (users). The vertices were then arranged using the Harel-Koren Fast Multiscale layout algorithm [65].

### Influential Voices of Each Network

Betweenness and outdegree centrality measures were used to determine the strength of relationships within the network and identify the top 10 users who were the most influential according to the social network theory [53]. Betweenness centrality identifies the center of each network and the users who have the greatest importance and “influence,” measured by the behavior and connectivity between the user and others within the network [30]. Degree centrality measures the number of unique connections linked to a vertex (user) and identifies the users that are “most popular” in the network [52,66]. In a directed graph, this is measured as in-degree (the number of connections directed toward the user, ie, another user posting about the user of interest) or outdegree (the number of connections directed away from the user, ie, user of interest actively posting) [52].

To identify the most influential “active influencers” (those who were actively posting and sharing information), the users with both the highest betweenness score and an outdegree score of at least 1.0, representing users who actively tweeted and were not only being “tweeted about,” were identified. These measures were used as users at the center of the network with the greatest number of connections served as a bridge connecting other users within each network. If these users were removed, the sharing and spreading of information would be affected [52].

Finally, user bio information and tweet content captured by NodeXL were used to categorize the accounts of the top 10 key users of each network (discussed earlier) and identify whether they actively supported the related popular diet. This was achieved by categorizing their account identity and identifying whether their tweets were suggestive of or actively promoting the diet to the general population.

### Interaction Between Popular Diet Networks

Text analysis metrics generated by NodeXL were used to identify the most common hashtags appearing in all tweets within each network [52]. This metric explored possible interactions between 16 popular diet networks by identifying common, recurring, and overlapping hashtags. Hashtags, as opposed to top words (also extracted by NodeXL), were used as they categorized and pinpointed key messages on precise topics [57-59].

### Interaction Between Networks and Mental Health

Text analysis was conducted using the NodeXL software to explore the potential associations between web-based popular diet networks and mental health. The NodeXL text analysis feature analyzes the words in each tweet and identifies whether the word is present in 1 of the 2 different word lists [52]. In the interest of our study, text analysis was used to explore the themes of mental health.

Two text classification word lists were created for (1) depression and anxiety and (2) eating disorders, based on the preexisting literature that used a combination of manual and lexical approaches [67]. Therefore, this process involved collating words from preexisting literature, which has demonstrated potential in measuring, classifying, identifying, and detecting various mental health conditions on social media [44-48,68-73]. The full list of text analysis words used can be found in Multimedia Appendix 2 [44-48,68-71,73].

To differentiate between each list, ensure accuracy, and comply with the NodeXL software [52], the following considerations were made when developing the lists: (1) removing all duplicate words from within each list, (2) ensuring that there were no words that overlapped between lists, and (3) removing words that appeared in the “stop” word list in NodeXL.

A sensitivity analysis was performed to account for words that related to popular diet keywords. For words considered in this analysis, refer to Multimedia Appendix 2. To account for words that overlapped between lists, the number of times that the word had appeared in the literature was considered (refer to Multimedia Appendix 3 for further details). If this did not promote a clear decision, a pilot test in NodeXL was used to identify the number of times that the word appeared in the corresponding network (Multimedia Appendix 3). In addition, as suggested by another study [68], superficially innocuous words such as “eating” and “exercise” were also excluded from the eating disorder list because of their potential ambiguity and inflation of results.

After both lists were created, a feasibility test was conducted to identify any additional words and symbols that needed to be added to the NodeXL “stop word” list. A number of words were identified and added if they had a word count of >100, under the premise that they would influence the salience (Multimedia Appendix 2). In addition, as “soy free” holds a different meaning in Spanish (soy free meaning “I am free”), Spanish was hidden from the “soy free” network analytics.

## Results

### Network Dissemination and How Messages Spread

**Figure 2:**
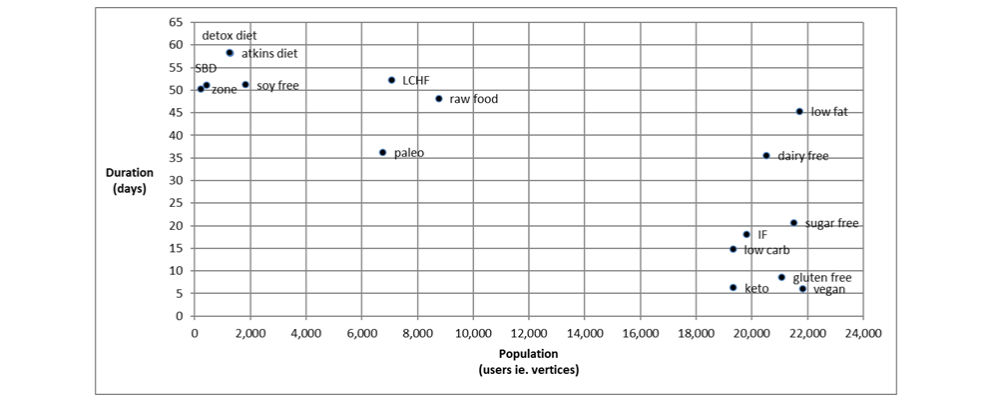
Scatter plot of the total population of each network over the time period of data collection. IF: intermittent fasting; LCHF: low-carbohydrate, high-fat; SBD: South Beach Diet; zone: zone diet.

The size and duration of each of the 16 networks are shown in Figure 2. Each network comprised all users and user interactions that occurred at the time of data extraction and has been labeled based on the popular diet being addressed. The vegan network had the largest network with data collected over the shortest duration (6 days 1 hour; n=21,819 users), followed by the ketogenic network (6 days 8 hours; n=19,336 users) and gluten-free network (8 days 14 hours; n=21,069 users).

Paleo (duration=36 days 7 hours; n=6750 users); raw food (duration=48 days 5 hours; n=8760 users); and low-carb, high-fat (LCHF); duration=52 days 6 hours; n=7060 users) had medium-sized networks over longer durations, whereas, Atkins diet (duration=58 days 4 hours; n=1283 users) and detox diet (duration=58 days 6 hours; n=1259 users) had smaller networks over long durations, with zone diet (duration=50 days 6 hours; n=211 users) and south beach diet (duration=51 days 2 hours; n=417 users) being the 2 smallest networks.

Visualization was created for all networks (Multimedia Appendix 4 [56]), which is a visual representation of all user interactions including tweets, mentions, and retweets. Overall, there were 2 dominant structures in all networks: complete fragmentation and hub and spoke. In most networks, the complete fragmentation structure was dominant, followed by the hub and spoke clusters. However, there was evidence of community interaction in the selected networks (keto, dairy free, intermittent fasting, vegan, and LCHF). LCHF was the only network where a hub and spoke structure was prominent.

### Influential Voices of Each Network

Of the 16 networks, 160 top 10 user accounts were identified through the betweenness centrality metric alone, with an additional 23 users after adjusting for the outdegree metric (N=183 users) and removing 10 duplicates (total n=173 users). User follower counts ranged from 4 to 72 million. A total of 8 users overlapped between the networks (Multimedia Appendix 5).

Using the information supplied in user bios, 11% (19/173) of the users were identified as claiming a health or science education, background, or profession, including medical doctors, MD (n=9; networks=paleo, low carb, keto, intermittent fasting, and LCHF), PhD, public health (n=1; network=paleo), dietitians (n=2; networks=Atkins diet and LCHF), nutritionists *(*n=3; networks=dairy free and zone diet), and other science education (n=4; networks=low carb, low fat, intermittent fasting, and LCHF). When considering the information collected from both user bios and tweets, 1 user appeared to actively oppose the diet (dietitian and Atkins diet), and 14 users actively endorsed the diet (paleo, vegan, low carb, zone diet, keto, intermittent fasting, and LCHF). LCHF (n=5 users) and intermittent fasting (n=3 users) contained the greatest number of health or science professional users endorsing the diet (Multimedia Appendix 5).

Of the top 10 user accounts, 31.2% (54/173) of the users across all networks openly supported the corresponding diet of the network they belonged to. Networks with the greatest number of active endorsers included LCHF (9/10, 90%), intermittent fasting (8/10, 80%), low carb (8/12, 67%), and keto (7/14, 50%). Networks with no endorsers were raw food, low fat, zone diet, and Atkins diet networks.

### Interaction Between Popular Diet Networks

**Figure 3:**
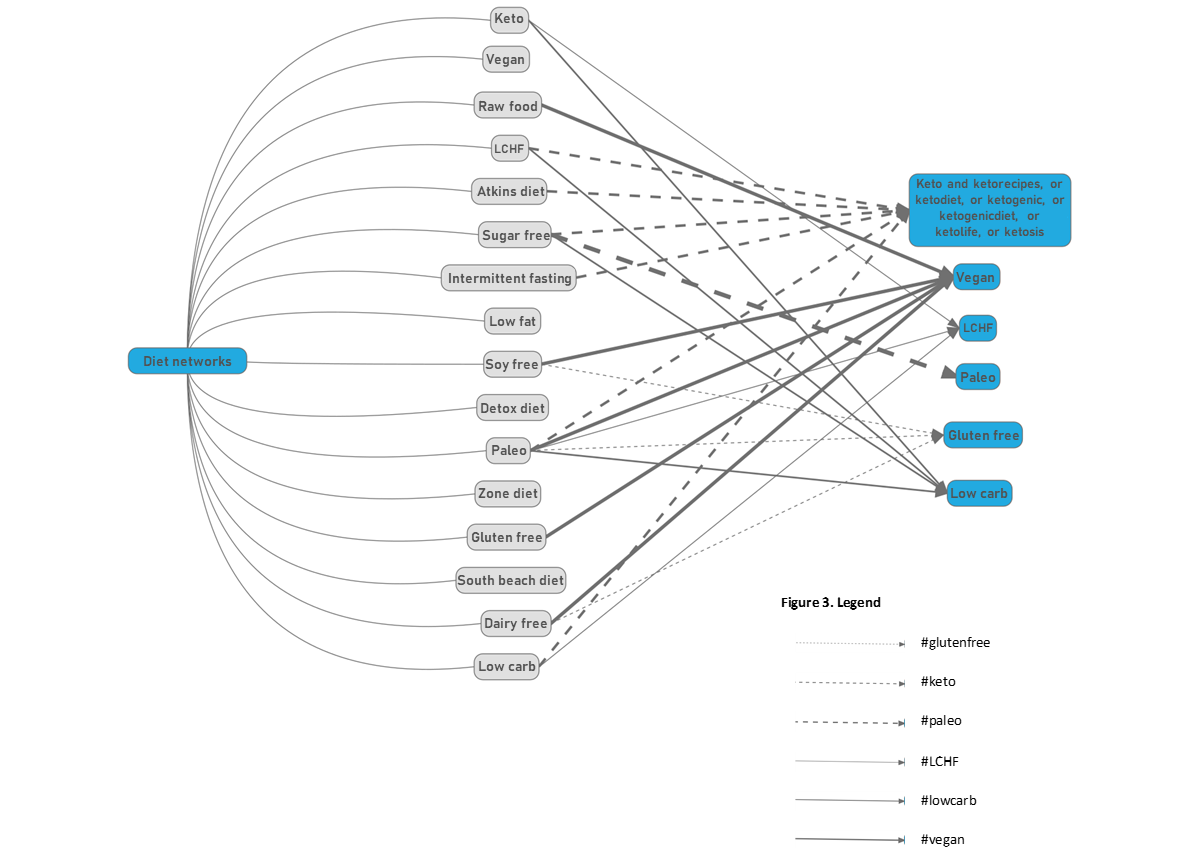
Popular diet (colored gray) network interactions as represented by top 10 hashtags (colored blue). LCHF: low-carbohydrate, high-fat.

The top 10 hashtags collected from all tweets within each network are presented in Multimedia Appendix 6. In total, 69% (11/16) of the popular diet networks (paleo, raw food, sugar free, dairy free, gluten free, low carb, Atkins diet, keto, intermittent fasting, LCHF, and soy free) displayed some form of interaction demonstrated by overlapping hashtags (Figure 3), although only 6 of the popular diets were referred to. The paleo network referenced the greatest number of diets, with 6 of its top 10 hashtags (keto, ketodiet, lowcarb, gluten free, vegan, and LCHF) representing 5 other networks. Keto had the greatest number of mentions across all networks (n=6; paleo, sugar free, low carb, Atkins diet, intermittent fasting, and LCHF). Of all the networks, LCHF referred to keto the most, with 7 of its 10 top hashtags. The vegan network made reference to 0 other diets.

Across all networks, #weightloss was the most prevalent hashtag used with 9 mentions (paleo, low carb, low fat, zone diet, Atkins diet, keto, intermittent fasting, detox diet, and LCHF), followed by #keto with 6 mentions. All diet networks made reference to at least 1 of the top-ranking hashtags except for the South Beach Diet that referenced 0 hashtags.

### Interaction Between Networks and Mental Health Frequency

Assessed using the content of all tweets within each network, depression and anxiety (words=113/3929; frequency: 0.029, 2.9%) and eating disorder (words=133/3929; frequency: 0.034, 3.4%) word frequency was the greatest in the “zone diet” network. In the depression and anxiety analysis, “zone diet” was followed by “Atkins diet” (words=493/24,760; frequency: 0.02, 2%), and word frequency was lowest in “soy free” network (words=240/43,874; frequency: 0.005, 0.5%). In the eating disorder analysis, “zone diet” was followed by paleo (words=6574/212,492; frequency: 0.031, 3.1%), and word frequency was equally the lowest in vegan (words=1521/446,955; frequency: 0.003, 0.3%), “dairy free” (words=1541/440,804; frequency: 0.003, 0.3%), and “gluten-free” networks (words=1578/451,842; frequency: 0.003, 0.3%). For all text analyses of mental health word lists, refer to Multimedia Appendices 5 and 7.

## Discussion

### Principal Findings

To our knowledge, this exploratory study is the first online SNA to explore and characterize the web-based discourse of popular diet networks. Our data provide novel insights into the dissemination of popular diet nutrition information on the web, key influential voices, interactions between web-based diet networks, and associations with mental health themes. The key findings of this study demonstrate that (1) i. social media activity reflects popular diet trends, and ii. nutrition information related to these diets is primarily dispersed through resharing information; (2) i. users claiming a background in science and health are among the most influential voices sharing nutrition information related to the popular diets explored, and ii. follower count does not necessarily affect influence on Twitter; (3) popular diet networks interact and connect through common dietary themes; and (4) there is evidence to suggest that a relationship may exist between popular diets and mental health in the context of web-based social networks.

### Network Dissemination and How Messages Spread

Popular diet trends change and evolve over time [21], and related dietary advice and information are regularly dispersed on the web [15]. Our results suggest that social media activity may reflect these trends, with the population and duration varying broadly for each of the diet networks. These findings indicate that each network is unique and imply that not every topic receives the same level of attention at the same point in time. In addition, it is plausible to assume that these dimensions will shift over time and that the size of the network may reflect diet popularity.

To our knowledge, there is limited longitudinal social media research exploring “popular” international diet trends over time; however, other research and Google worldwide trends demonstrate that the zone and Atkins diet were at their peak of popularity in the 1990s [21], the South Beach Diet in 2004, and the detox diet in 2007 [74], all of which have smaller networks. Larger networks, including vegan and keto, hit their peak in 2019, and gluten-free network has remained popular since 2013 [74]. In addition, the International Food Informational Council Food and Health Survey, released June 2020, found that keto and intermittent fasting were among the top 3 most common diets followed at this time, with low-carbohydrate and gluten-free diets following closely behind [75]. Conversely, despite being our largest network, the vegan diet was reported as 1 of the least common [75], which may reflect ethical motivations for veganism as a “lifestyle” rather than a diet trend [76,77]. Conversely, larger diet networks that seemingly never had a “peak,” such as sugar free [74], may predict the emergence of new popular diets. They may also reflect common keywords spanning a number of networks, or they may simply be representative of dietary descriptors and ethos (eg, sugar-free recipes). In addition, smaller and medium-sized networks may reflect trends that have passed, or keywords may not be representative of the way the diets are discussed on the web. The results of this study highlight the role of Twitter in dispersing nutrition information on the web, with larger networks allowing for greater amplification and dissemination of dietary messages. It also demonstrates that messages on social media can continue to spread even decades after they were most relevant.

The structures that emerged through visualization provided additional insights into the dissemination of information [51]. The dominant structure suggests that most users were disconnected and talking about the diet rather than talking *to* each other. Practically, this indicates that influencers were getting retweeted by users without connecting through mutual conversation. To our knowledge, there are currently no network analyses of popular diet networks on Twitter; however, a SNA of the social media platform—Reddit found that users formed close relationships with dense interactions in 3 weight loss–focused networks [78]. Comparisons should be made with caution; however, owing to the presumed connectedness of social media [79], a similar observation was expected from our results. With this in mind, LCHF supported this assumption, dominated by a hub and spoke structure, suggesting a more complex community interaction. Interestingly, LCHF was selected through feasibility testing rather than academic literature. This finding suggests that web-based communities develop their own language to connect on the web. This concept may be similar to that of eating disorder communities, where 1 study found that users connected through specific hashtags [68].

These findings provide valuable insights into the dispersal of “popular” diet-related information on the web and suggest that nutrition professionals could create an impact by resharing key information from within the health community. Further research on the application of resharing nutrition content could inform strategies as part of community and government initiatives in the future. In addition, to gain a deeper understanding, we recommend that future social network analyses and longitudinal research explore how web-based diet networks change over time.

### Influential Voices of Each Network

Information dissemination was affected by the most influential voices of each popular diet. In accordance with the social network theory, they form the center of the network and act as a mediator of information between users [7,51]. Our findings identified regular Twitter users, businesses, brands, and users claiming a background in science and health among the most influential endorsers of popular diets, all having a considerably different follower count. This result was somewhat unexpected, with the follower count of other social network platforms a determination of influence [80]. However, this finding is similar to that of a study that explored user influence on Twitter, which found that retweets and mentions exhibited more influence than followers [81].

Furthermore, to the best of our knowledge, no research has identified the key influential voices promoting popular diets on the web. Studies that explore the web-based communication of health messages may provide some insights into their position within the network. With this in mind, studies have found that heroic language increases perceived authenticity [82], and “experts” are perceived as more trustworthy [83]. Although direct comparisons cannot be made, this may be because of the language used by users and perceived expertise as contributing factors for posts being reshared. Public trust in “experts” [84] may have contributed to our findings, where 11% (19/173) of the top influencers identified as claiming a background in science or health. Notably, most of these top “health and science” influencers endorsed the corresponding diet, this included only 2 dietitians, one of whom was actively opposing the popular diet (speaking out about misinformation) and another who was an academic researching the popular diet (Multimedia Appendix 5). Although we did not assess the validity of the diet claims made in this study, the endorsement of these diets and the contribution of nutrition-related information from unqualified users must be acknowledged. This may also reflect a study that conducted an analysis of health-related tweets and found that more than half were not evidence based [85]. It would be beneficial for future research to assess and validate the reliability of tweets by both health professionals and unqualified users.

Finally, it must also be acknowledged that the data collected from the popular diet communities may not reflect those of professional networks. However, our findings identify the need for more qualified nutrition professionals, such as dietitians, to gain a presence on the web. To encourage social media use and overcome potential barriers [30], educational institutions and professional organizations, such as Dietitians Australia, the Association of UK Dietitians, and the Academy of Nutrition and Dietetics, could expand their media training to include more comprehensive social media training. This may also be extended to other health professionals to encourage its use in a meaningful and safe way. In addition, monitoring how key influencers may change over time and a content analysis of tweets may provide insights into user motivation.

### Interaction Between Popular Diet Networks

Associations between various popular diets may be observed through the exploration of top hashtags, which are commonly used to ascertain related themes [58]. These themes were evident in our study, with networks connected through shared dietary ethos and topics such as weight loss and health. To our knowledge, there is currently no literature that explores web-based interactions between various diet networks; however, popular diets, such as fad diets, are often categorized by their distribution of macronutrients [21]. Although direct comparisons cannot be made, this concept may provide insight into our results; interestingly, although 69% (11/16) of the networks interacted, only 6 were referred to, most of which were characterized by a high-fat, low-carbohydrate eating pattern [86,87]. Notably, however, with the exception of the vegan and gluten free networks, which may reflect veganism as both a fad and ethical approach to eating [76,88] or gluten free as another way to describe a low-carbohydrate eating pattern, or as an allergy and dietary descriptor (eg, gluten-free bread) [87].

In addition, and as explored earlier, dietary interaction may also reflect trending diets, with keto and vegan being the most referenced [74]. Our findings suggest that popular diets interact on the web and connect through similar themes and ethos. Identifying these connective themes may assist dietitians and nutrition professionals in directing their messaging and addressing potential misinformation being shared on the web.

### Interaction Between Networks and Mental Health

As web-based diet content can influence food choice [27] and social media can provide an “echo chamber” of similar information, [24] exposure to web-based diet content may lead to unnecessary restriction by those consuming the information. Restrictive diets have also been linked to potentially negative physiological and psychological health outcomes including eating disorders and depression and anxiety [20,21]. Therefore, this study explored the themes of mental health and their interaction with web-based diet networks. Despite these known associations, to the best of our knowledge, there is currently no literature exploring web-based mental health word frequency within popular diet networks. Although word frequency must be interpreted with caution owing to the complexity of social media [89], related research has identified the potential for detecting mental health symptoms on the web [46,68]. With this in mind, the results of our study identified several networks where words suggestive of mental health concerns were present.

The novel nature of this exploration prevents a deeper interpretation of these findings; however, from a social media perspective, identifying communities where mental health narrative is strongest may allow social networks and organizations to target specific mental health–related support or relevant advertisements (such as mental health–specific helplines) to reach the people that need it most. This concept was explored in a mixed methods survey, where 60% (96/160) of participants endorsed the idea of using social media technology to improve targeting of mental health services [90]. Although it must be interpreted with caution, our results suggest targeting “web-based diet communities” that promote eating patterns that contain rules about carbohydrate intake, with support specific to depression, anxiety, and eating disorders. From a nutritional perspective, although there may be some association between mental health word frequency and specific diet patterns, direct comparisons cannot be made. However, our results reflect what is known in the current dietary literature, where mental health word frequency was found to be highest in a diet with rules about carbohydrate intake [20,21], whereas diets rich in carbohydrate-containing foods, such as whole grains and vegetables, are associated with beneficial effects on mental well-being [91]. Similarly, eating disorder word frequency was found to be the highest in 2 dietary patterns governed by a set of rules and restrictions, which may also correspond with eating disorder symptomology [92,93].

Although these results must be interpreted with caution, these novel insights are suggestive of an existing interaction between popular diet networks and mental health. More in-depth exploration is needed in future research to improve our understanding of this finding, including the directionality of the relationships and the importance of the word frequency. Content analysis of tweets and survey research should also explore consumer experiences and the perceived influence of social media on food choice. Network analysis research may also be used to identify topics that mental health consumers engage with on the web, and in the future, results may help to inform targeted web-based diet and mental health–related initiatives.

### Limitations

Some additional limitations of this research must be considered, including (1) using Twitter posts only, (2) the removal of duplicate keywords from text word lists (Multimedia Appendices 2 and 3) that may have resulted in lower word frequency than presented, (3) errors of omission because of analyzing data from only 1 period, and (4) unknown gaps in data owing to access challenges such as privacy settings.

### Conclusions

In conclusion, our study provides novel insights into the web-based discourse of popular diet networks and has paved a way for similar research in the future. Our findings are important for health professionals and related organizations to enhance their understanding of the web-based nutrition space and to help inform the effective dissemination of nutrition messages by qualified professionals on the web. To build on our findings, further network analysis and longitudinal and survey research are needed to explore popular diet trends on the web over time and to understand the impact that social media can have on dietary choices. Finally, to encourage qualified nutrition professionals, such as dietitians, to be the leading voices of nutrition information on the web, we recommend social media training for health professionals and that dietitians and nutrition professionals work together as a community by actively resharing posts.

## Appendix

Multimedia Appendix 1 Top 10 hashtags, network metrics, and figure visualizations.

Multimedia Appendix 2 Text analysis of mental health words list.

Multimedia Appendix 3 Mental health word list frequency and overlapping words.

Multimedia Appendix 4 Graph visualization.

Multimedia Appendix 5 Results summary table.

Multimedia Appendix 6 Top 10 hashtags.

Multimedia Appendix 7 Text analysis for mental health word lists.

## Abbreviations

API: application programming interface
LCHF: low-carb, high-fat
SNA: social network analysis
