# Supplementary material for: Characterizing the Discourse of Popular Diets to Describe Information Dispersal and Identify Leading Voices, Interaction, and Themes of Mental Health: Social Network Analysis
Source: JMIR Infodemiology. 2023 May 5;3:e38245. doi: 10.2196/38245 (PMC10199384; doi:10.2196/38245)
Supplement: Multimedia Appendix 1 [file infodemiology_v3i1e38245_app1.docx]

**Appendix I - Methods**


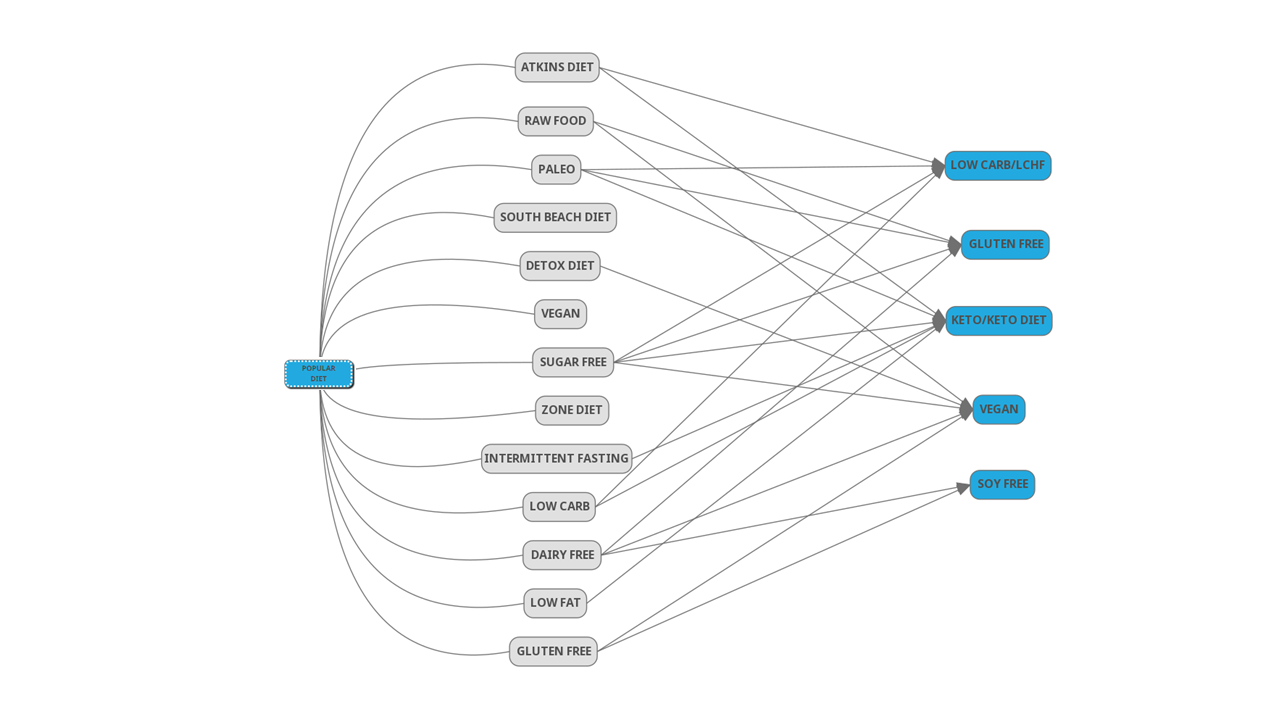


Figure 1. Network map of top 10 hashtags representing popular diet networks. (Diet network search words are colored grey. Top 10 hashtags are colored blue).

Table 1. Network metrics definitions [52].

| **Network metric** | **Definition** |
| --- | --- |
| Vertices | Otherwise known as a vertex, nodes, users, entities or people. The vertices count is the number of people or things in the network. |
| Edges  Unique edges | Otherwise known as a relationship, tie or link. An edge represents the connection or interaction between two vertices (users).  When a connection between A and B (two unique users) are online counted once. |
| Cluster algorithms | Identify, group, and analyse network vertices (users) that have shared characteristics. |
| Graph visualisation | A picture of thesocial relationships amongst users within the network. |
| Centrality measures  Betweenness  Degree  In-degree  Out-degree | Determine the strength of relationships within the network.  The people at the centre of the network.  The number of unique edges connected to a vertex (user).  The number of edges that point towards a vertex (user).  The number of edges that the vertex (user) points towards. |
| Time series | The time frame of activity in the network, within the collected data set |


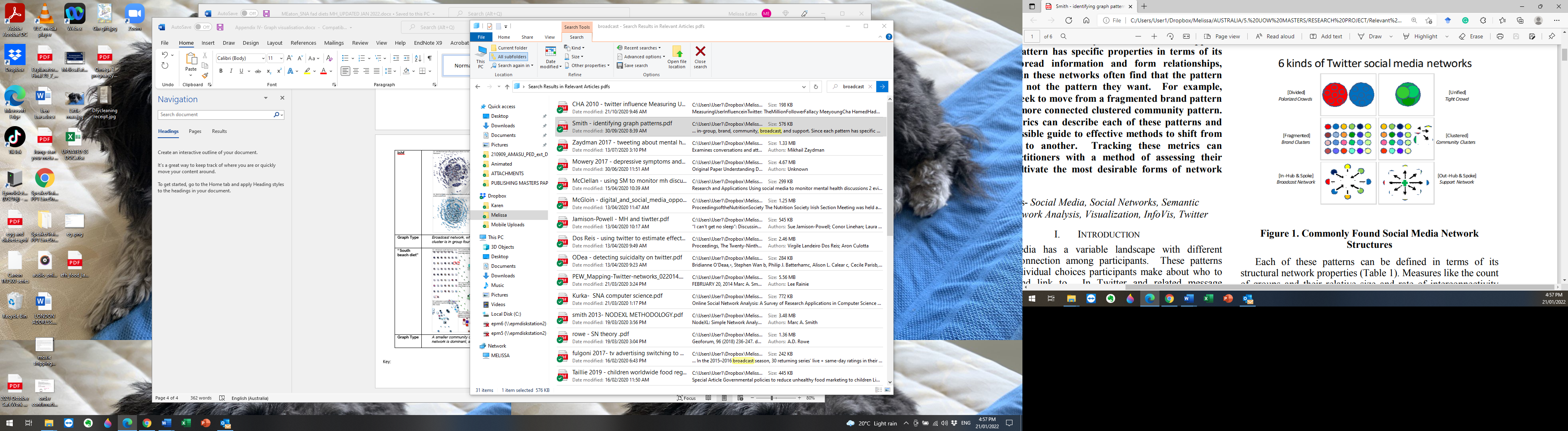


Figure 1. Figure structure visualizations [56].
