# Supplementary material for: Characterizing the Discourse of Popular Diets to Describe Information Dispersal and Identify Leading Voices, Interaction, and Themes of Mental Health: Social Network Analysis
Source: JMIR Infodemiology. 2023 May 5;3:e38245. doi: 10.2196/38245 (PMC10199384; doi:10.2196/38245)
Supplement: Multimedia Appendix 2 [file infodemiology_v3i1e38245_app2.docx]

**Appendix II – Text analysis of mental health words list**

**DEPRESSION & ANXIETY**

| **Code** | |
| --- | --- |
| **Bold** | Words similar to popular diet keywords, for sensitivity analysis |
| Underlined | Words also appearing in the Eating Disorder list |

**Note: duplicates between studies have been removed**

**De Choudhury et al 2013 - Social Media as a Measurement Tool of Depression in Populations**

depressed depressive depression depressants depressant depressing depressingly depressions weak life blame problems torture safe escape worry uncomfortable shit pressure conversation myself worth break nobody mine painful hate intimidating intimidate intimidated intimidates suck sucks

**De Choudhury et al 2013 – Predicting Depression via Social Media.**

anxiety withdrawal severe delusions adhd weight drowsiness suicidal appetite dizziness nausea episodes attacks sleep seizures addictive weaned swings dysfunction blurred irritability headache imbalance nervousness psychosis drowsy medication side-effects doctor doses effective prescribed therapy inhibitor stimulant antidepressant patients neurotransmitters prescriptions psychotherapy diagnosis clinical pills chemical counteract toxicity hospitalization hospitalisation sedative 150mg 40mg drugs

**Mowery 2016 - Towards Automatically Classifying Depressive Symptoms from Twitter Data for Population Health**

alone cried cries crushed cry grief lose loses losing lost pain sad sobbed sobbing sobs unimportant wept whining insomnia tired abandon abandoned abandon abandonment abandoning abandons ache aches ached aching agony agonising agonizing agonies agonise agonize agonized agonised agonises agonizes agonisingly agonizingly broke brokenness broken brokenness damage damaged damaging damages damagingly defeat defeated defeating deprive depriving deprived deprivation deprival deprives despair despaired despairing despairs devastating devastated devastation devastative devastates devastate disadvantage disadvantaged disadvantages disappoint disappointed disappoints disappointing discouraged discouraging discourager discourages discourage dishearten disheartening disheartened disillusion disillusioned dissatisfy dissatisfied dissatisfies dissatisfying doom doomed doomsaying doomfully doomsters doomsayer doomster dull dulls dullness dullish dullishly dullest dulling duller dulled dully dullnesses empty emptied emptiness empties emptinesses gloom gloominess gloomiest gloomings glooming gloomy gloomful gloomier gloomily gloomed glooms grave graved graven grieve grieving grieves grievance grievances grievously grevious grim grimly grimmer grimace grimaces grimness grimmest grimaced grimacing grimalkin grimy griminess grimnesses fail fails failed failure failing failings failures failingly flunk flunks flunked flunking heartbreak heartbreaks heartbreaking helpless helplessly helplessness homesick homesickness hopeless hopelessness hopelessly hurt hurts hurting hurtful hurtfulness hurtfully hurtful inadequate inadequacy inferior inferiority inferiors inferiorly isolate isolatable isolation isolated isolating isolates isolator lame lamer lamest lone loner lonely loners lonelier lonelily loneness lonesome lonliest lonesomes loneliness lonenesses lonesomely longing longingly longings loser losers loss losses melancholy melancholia melancholic misery miserable miserably miseries mourn mourns mourner mourned mournful mourning mourners mourning mourningly mournfully mournfuller neglect neglects neglecters neglectful neglecting neglectors neglected neglecter neglector neglecting overwhelm overwhelms overwhelming overwhelmed pathetic pathetical pessimist pessimists pessimistic pessimism pitiful pitifulness pitifuller pitifully pity pitying pityingly regret regrets regretful regretted regretter regretting regretters regrettably regrettable regretfully reject rejects rejected rejector rejectee rejecter rejection rejections rejectingly remorse remorseless remorseful remorses resign resigns resigned resigner resigners resigning resignedly resignation ruin ruins ruing ruined ruiner ruinous ruinate ruiners ruining ruinates ruinated ruinable ruinously ruination ruinating ruinations ruinousness solemn solemnly solemnity solemnifies solemnifying solemnified solemner solemnest solemnness solemnify sorrow sorrows sorrowfully sorrowers sorrowful sorrowing sorrowed sorrower suffer suffers sufferer suffered sufferings sufferance sufferably sufferable sufferances tear tears tearstained tearstains tearstain tragedy tragedies tragic tragics tragically tragical unhappy unhappiness unhappiest unhappier unhappily unsuccessful unsuccessfully unsuccessfulness useless uselessness uselessly weep weeps weepy weeper weeping weepies weepier weepers weepiest weepiness whine whiney whines whiner whined whiners woe woes woeful woeness woesome woefully woenesses woefuller woebegone woefulness woefullest worthless worthlessness worthlessly yearn yearns yearned yearner yearning yearners yearnings yearningly fatigue fatigues fatigued fatiguing fatiguingly

**Mowery et al 2017 - Understanding Depressive Symptoms and Psychosocial Stressors on Twitter: A Corpus-Based Study**

crying insomniac guilt guilty concentrate focus indecisive killl suicide fired unemployment homeless

*Note:* Words are part of a “31 word” list (duplicates have been excluded) identified by Mowery et al 2017 as being the most informative for classifying depressive symptoms.

**Kumar et al 2019 - Anxious Depression Prediction in Real-time Social Data**

**Fat** bad problem illusion restless crap fuck meaningless crying sleepless never bored afraid ugly upset awful suffer nobody shatter kill panic frustrated destroy die sick

**Shen & Rudzicz 2017 – Detecting anxiety on Reddit**

anyone social friends feeling having anxious talk thought better felt my;anxiety social;anxiety my;life anxiety;and anxiety;I anyone;else talk;to panic;attacks panic;attack where;I self;esteem side;effects mental;illness heart;rate x;post mental;health physical;symptoms social;media hey;guys does;anyone;else thanks;for;reading no;matter;how wondering;if;anyone having;panic;attacks stop;thinking;about in;high;school get;rid;of has;anyone;else wanted;to;share

**EATING DISORDER**

| **Code** | |
| --- | --- |
| **Bold** | Words similar to popular diet keywords, for sensitivity analysis |
| Underlined | Words also appearing in the Depression & Anxiety list |

**Note: duplicates between studies have been removed**

**Zhou et al 2019 - Analysis of Twitter to Identify Topics Related to Eating Disorder Symptoms.**

EDproblems anamia anorexia anorexic bodyslip bonespo bulimia bulimic chestbones collarbones edlogic ednos edprobs proana promia thinspiration thinspo thinspos calorie slim bone chest hip thigh hipbone skinny stomach flat body;slip body;check selfie weight;loss **diet** recovery therapy treatment worth plan cry hungry ill starve failure mirror damn bony under;weight sad anorex feel gainsomeweight skinnyleg thighgap bikinibridge ana projectthin weightloss eatingdisorder anger edproblem EDfamily AnxietyProbs

**Cavazos-Rehg et al 2019 – *“I just want to be skinny.”* : A content analysis of tweets expressing eating disorder symptoms**

hip;bones hipbones pro;ana chest;bones pro;mia

**Wang et al 2017 – Detecting and Characterizing Eating-Disorder Communities on Social Media**

eating;disorder anorexia;nervosa bulemia bulimia;nervosa edprob askanamia purge binge legspo Body;Mass;Index Current;Weight Ultimate;Goal;Weight Goal;Weight HW Highest;Weight Lowest;Weight lbs kg

**Arseniev-Koehler et al 2016 – #Proana: Pro-Eating Disorder Socialization on Twitter**

Pro-ED pound RG mia miaana proed osfed wannarexic wannarexics wannarexia edproblems edtruths edlife anasisters anabuddy ED disordered;eating overweight **fat** obese fatty fattest whale skinniest skinnier thin thinner skeleton emaciated ribs backbone bones collarbone thighs hips belly weight;gained scale scales gw cw lw ugw bmi pounds kgs weighed calories caloric cals skinnyplease starved starving hunger skip fasted **fasting** throwing throw threw vomit vomited vomiting throwup puke puked puking purged purging binged binging bloat bloated lax laxes laxies laxatives laxative CalorieApril ProjectThin Thinspo Bonespo Thinspiration Perfection perf rg Russian abc abcdiet

**Did not include in analysis:**

**Zhou et al 2019 - Analysis of Twitter to Identify Topics Related to Eating Disorder Symptoms.**

Amazing fit workout gym vitamin fridge entire daily stay night anything old think nothing hot fitness perfect pepperoni pizza everything

**Arseniev-Koehler – #Proana: Pro-Eating Disorder Socialization on Twitter**

food breakfast dinner meals eating eat ate appetite perfect workout abs jog elliptical exercise miles gym treadmill

**Text analysis of mental health words list**

**‘STOP WORDS’**

**NodeXL Default**

0 1 2 3 4 5 6 7 8 9 a à â å ä ã ab aber able about across after ahora ain't al all almost als also am among an and años any aqui aquí are aren't as así at au auch auf aunque aus avec b be because been bei beim bin bis but by c cada can can't cannot che com como con could could've couldn't cuando d ð da damit dann dans das dass de dein deine deinem deinen deiner deines del della dem den denen denn der deren des desde después dessen di dich did didn't diese diesem diesen dieses dijo dir do doch does doesn't don't donde dort dos du durante durch ðÿ ðÿš e é è een een ein eine einem einen einer eines either el él ella elle else en entre er era es esa ese eso est esta está este esto estos et etwa etwas euch euer euren eures even ever every f for för from fue für g get gleich got h ha haben había hace had han has hasn't hasta hat hatte hätte hatten hätten hättest have hay he he'd he'll he's her here hers het hier him his how how'd how'll how's however http https i ï i'd i'll i'm i've ich if ihm ihn ihnen ihr ihre ihrem ihren il im in ins into is isn't ist it it's its j je jetzt just k kann können konnte könnte konnten könnten konntest könntest konntet l la las le least les let like likely lo los m más may me mein meine meinem meines menos mi mich might might've mir mismo mit moi most muss musste müsste mussten müssten müsstest must must've mustn't muy my n ñ na nach ne neither new ni nicht no noch nor nos not nous now nun nur o ó ö ob och oder of off often om on only op or other otra otro otros ou our out own p på page pages país para parte pas per pero por porque post posts pour puede q que qué qui quoi r rather real rt s said say says schon se según sehr sein ser she she'd she'll she's should should've shouldn't si sí sich sido sie siempre sin since sind so sobre sogar soll sollst sollte sollten solltest solo sólo som some son sous soy ß su sur sus t también tan tanto te than that that'll that's the their them then there there's these they they'd they'll they're they've this though through tiempo tiene tja to todo todos toi too tres tu tun u ü über um un una und une uno uns unser unsere unserem unseren unseres unter up us v va van vez vi via vía vom von voor vor vos vous w want wants wäre wären wärest was wasn't we we'd we'll we're wenn wer werden were weren't what what's when where where'd where'll where's which while who who'd who'll who's whom why why'd wie will wir wird wirst with wo won't would would've wouldn't wurde wurden würden wurdest würdest www y ya yet yo you you'd you'll you're you've your z zu zum zur

**Added based on feasibility test**

amp baru gue aja yang ada jadi tau siapa kalian _ ม ง ไม ส ด ค ผ ก san من บ ย ร หร อย เช buat และม งในแง เก aku ako sa น न ल dian र गर क ह स म त य ट द छ ब प të फ ˆà don ว เด อน ทำ ำ นในช งและน ะ โล จากน ตอนน งส ดค não pra mas ซ งถ minha só faz ท วย เน านฉ ãƒ æ ç ฮ วฟ กㅠ ªã ø ì ÿã ù ë จ ____ šã คร ˆã tapi kalo ga เป atau ini tak itu อร mau suka ê กท µà šà ƒã kalau dal nei yung bakit ข ต ถ า วส เร ara anda d í dalam kini itâ œ iâ ªà tem î dar elas vai œã non žà ll อ œà œðÿ wh pe
