# Supplementary material for: Characterizing the Discourse of Popular Diets to Describe Information Dispersal and Identify Leading Voices, Interaction, and Themes of Mental Health: Social Network Analysis
Source: JMIR Infodemiology. 2023 May 5;3:e38245. doi: 10.2196/38245 (PMC10199384; doi:10.2196/38245)
Supplement: Multimedia Appendix 3 [file infodemiology_v3i1e38245_app3.docx]

**Appendix III – Mental health word list frequency, overlapping words**

| **Words that appeared in both MH word lists** | **Number of times appeared in various DEP & ANX literature** | **Number of times appeared in various ED literature** | **Decision** |
| --- | --- | --- | --- |
| **Therapy** | I + 1 word including therapy | I + 1 word relating to treatment | - |
| **Worth** | I + 4 words starting with worth | I | ANX/DEP |
| **Cry** | III + 2 words starting with cry | I | ANX/DEP |
| **Failure/s** | III | I | ANX/DEP |
| **Sad** | III | I | ANX/DEP |
| **Weight** | I | III + 15 words that contain weight | ED |
| **Fat** | I | I + 3 words that contain fat | ED |

ED = Eating Disorder, Dep = Depression, ANX = Anxiety

**NodeXL pilot test for *therapy*:**

At a rate limit of 1,000 tweets, data was collected for the words “eating disorder”, “depression” and “anxiety” in July 2020.

For ED, “therapy” appeared six times in 1,000 tweets, and four times as part of a word pair.

For DEP, “therapy” appeared 7 times in 1,000 tweets, and 12 times as part of a word pair.

For ANX, “therapy” appeared 11 times in 1,000 tweets, and 14 times as part of a word pair.

Therefore, “therapy” will be used for the DEP and ANX sentiment.
