# Supplementary material for: Characterizing the Discourse of Popular Diets to Describe Information Dispersal and Identify Leading Voices, Interaction, and Themes of Mental Health: Social Network Analysis
Source: JMIR Infodemiology. 2023 May 5;3:e38245. doi: 10.2196/38245 (PMC10199384; doi:10.2196/38245)

**Appendix IV – Graph Visualisation**

Table 1. Graph visualisation of the 16 popular diet networks

| **Keyword**  **(Diet)** | **Graph Image** | **Keyword**  **(Diet)** | **Graph Image** |
| --- | --- | --- | --- |
| **Paleo** | 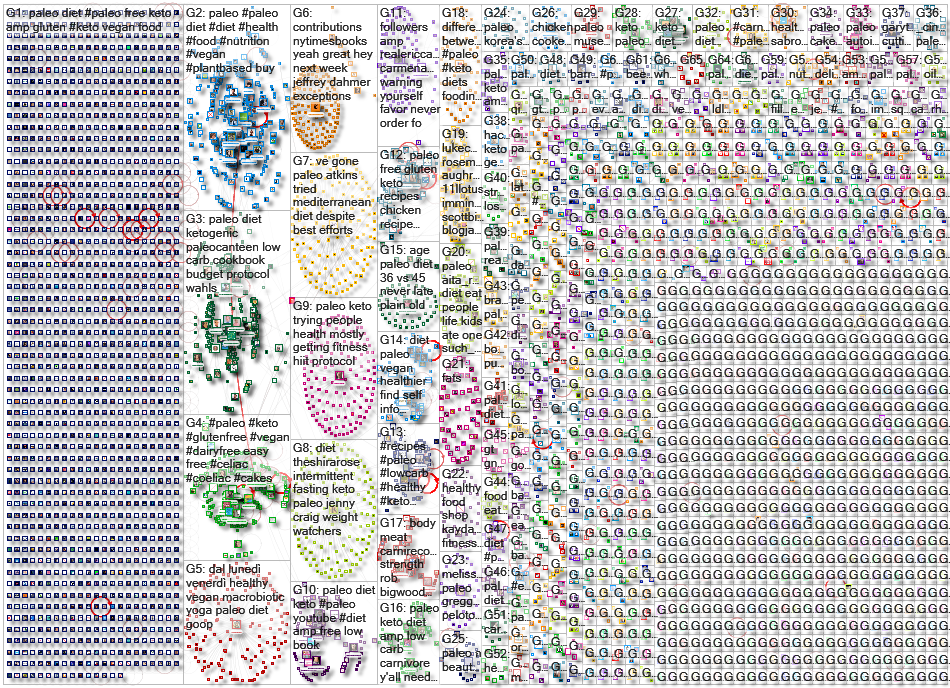 | **"zone diet"** | 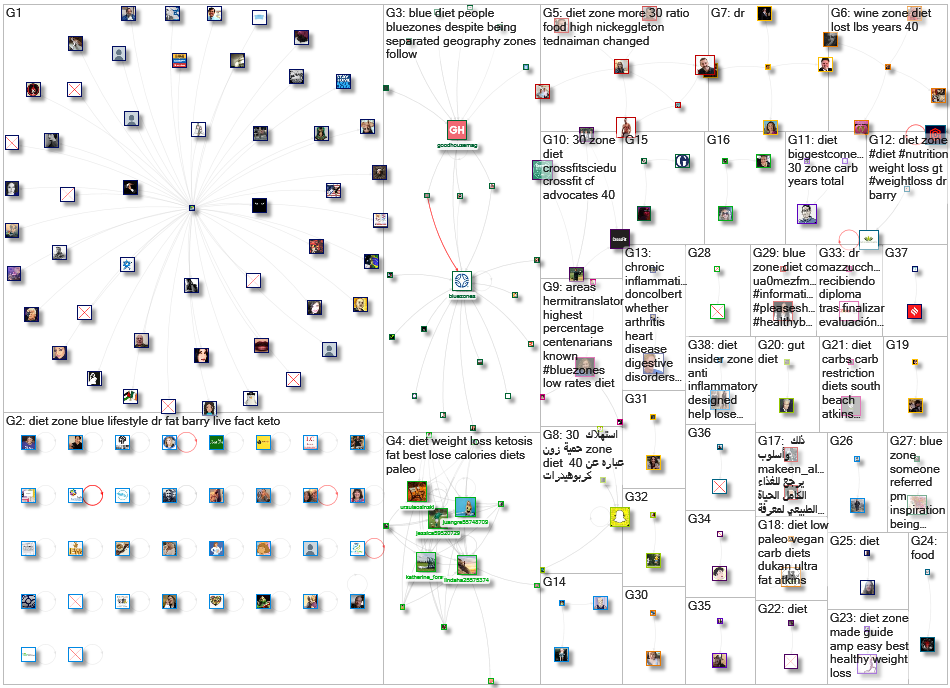 |
| **Graph Type** | *Community cluster network, where brand cluster is dominant and all other clusters are broadcast.* | **Graph Type** | *A smaller community cluster network where the broadcast network is dominant, followed by brand cluster, and all remaining are broadcast.* |
| **"raw food"** | 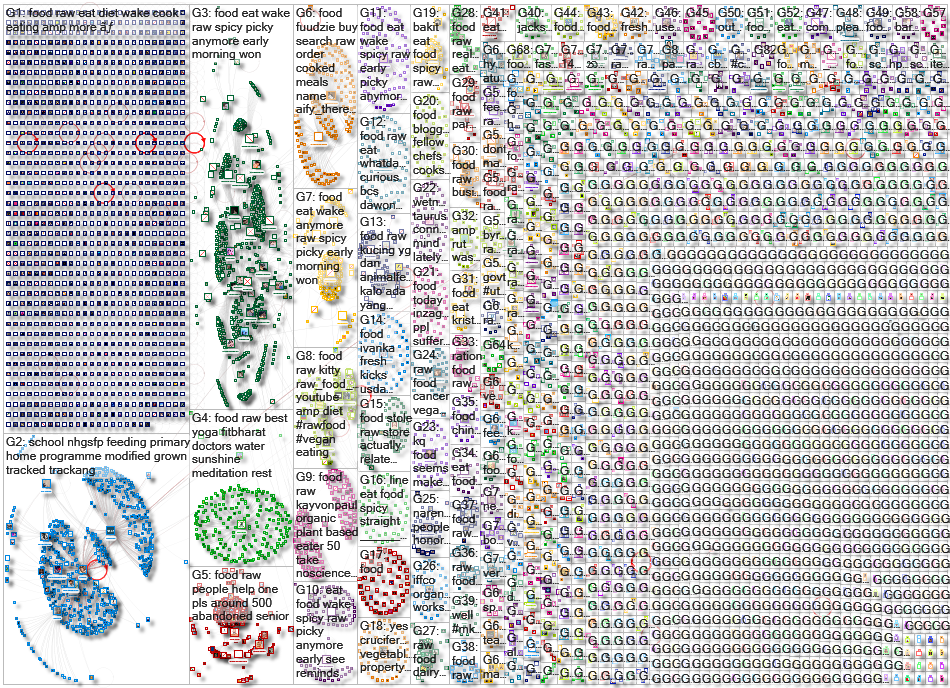 | **"atkins diet"** | 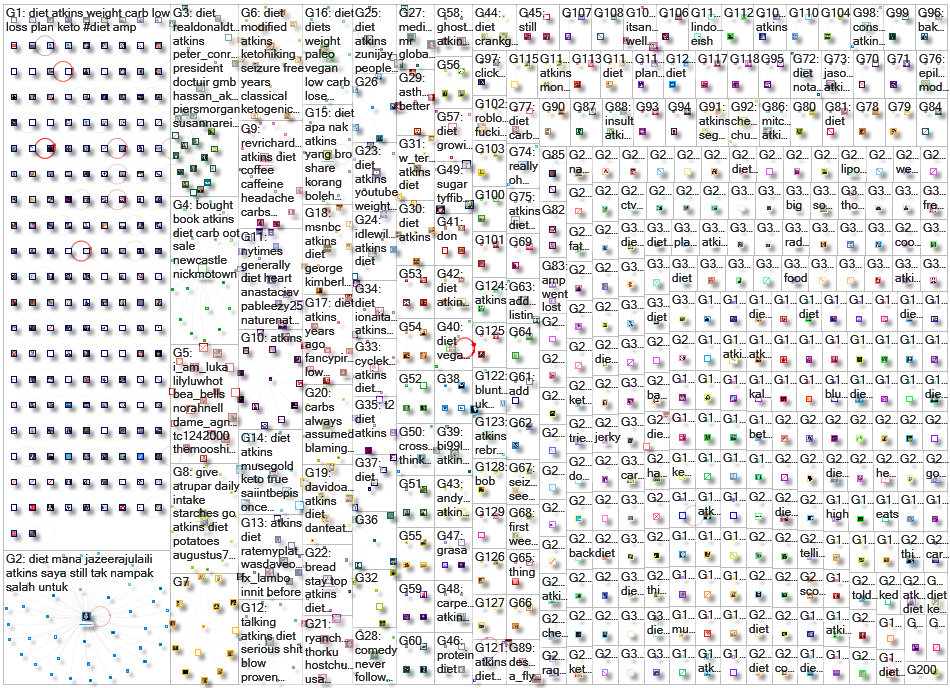 |
| **Graph Type** | *Community cluster network, where brand cluster is dominant and remaining groups are broadcast.* | **Graph Type** | *Dominated by a brand clusters with the remaining clusters being broadcast.* |
| **"sugar free"** | 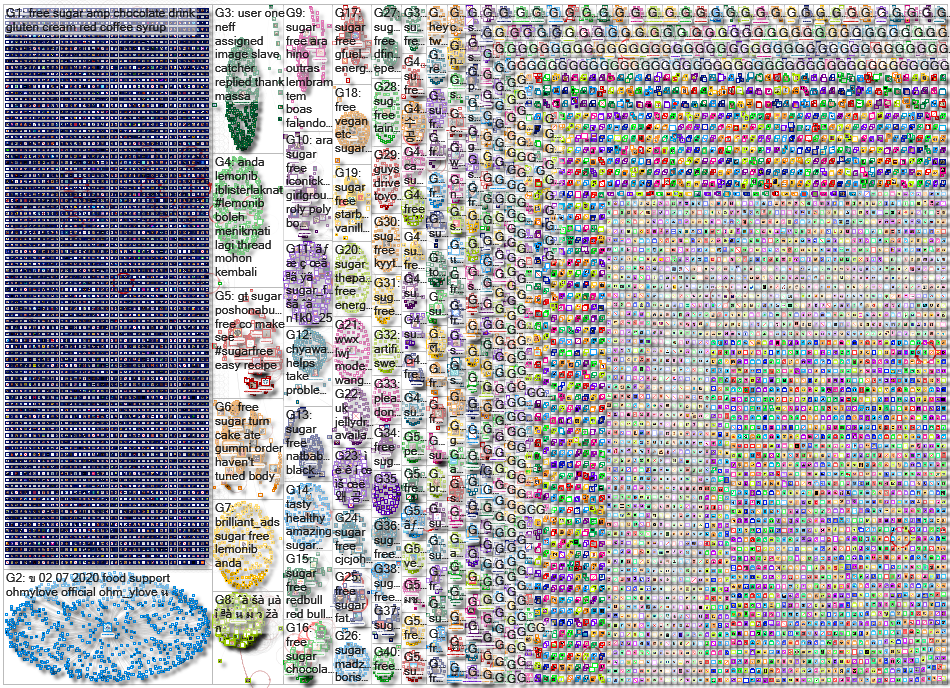 | **keto** | 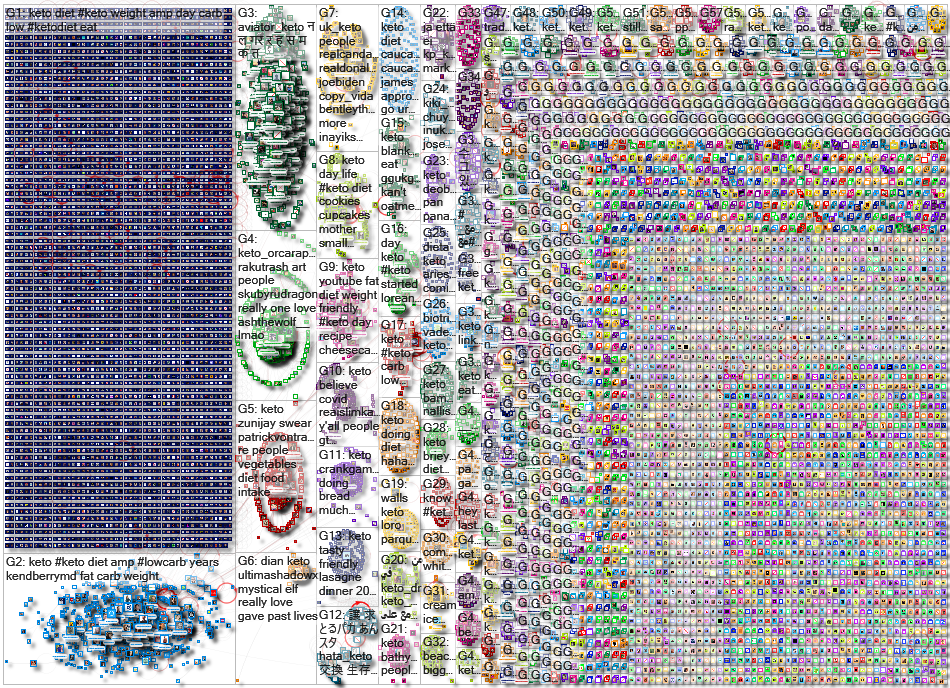 |
| **Graph Type** | *Community cluster network, where brand cluster is dominant and all other clusters are broadcast.* | **Graph Type** | *Community cluster network, where brand cluster is dominant, all other clusters are broadcast and there is possible evidence of community interaction in group 2 and 3.* |
| **"gluten free"** | 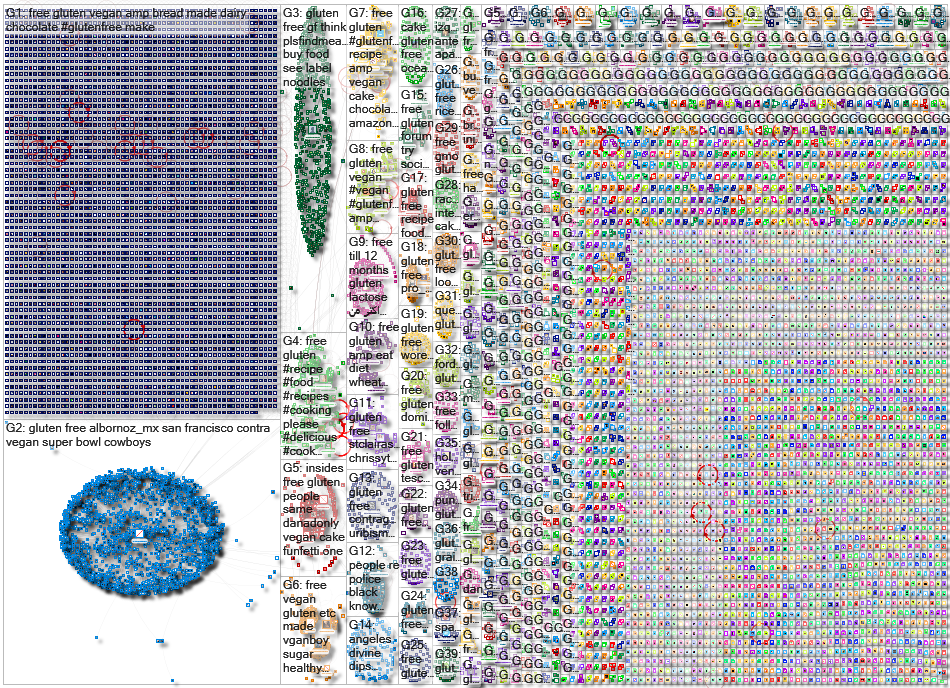 | **"detox diet"** | 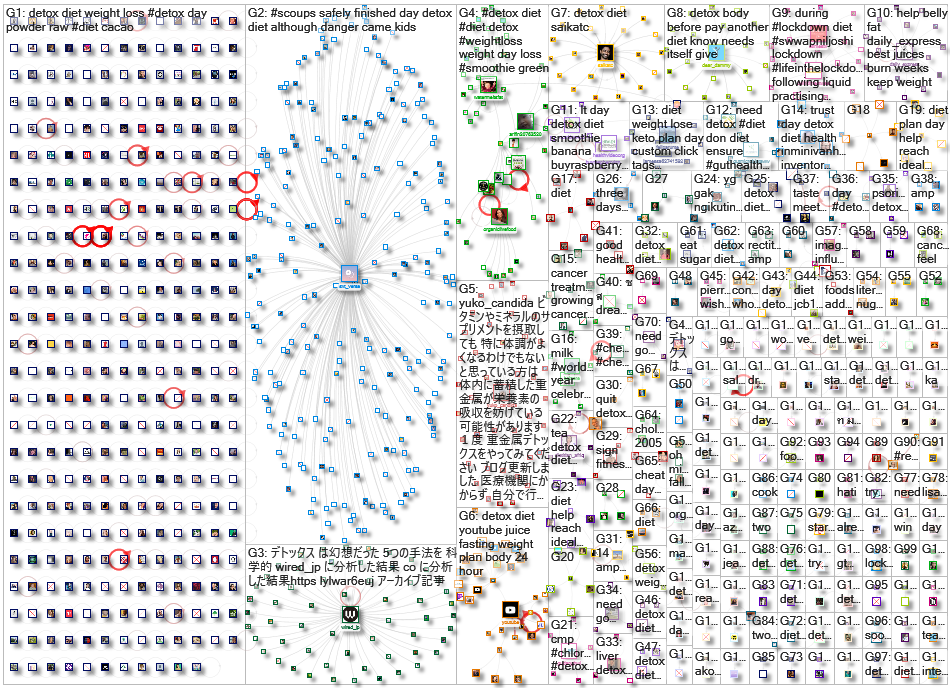 |
| **Graph Type** | *Community cluster network, where brand cluster is dominant and all other clusters are broadcast.* | **Graph Type** | *Community cluster network, where brand cluster is dominant and all other clusters are broadcast.* |
| **"low fat"** | 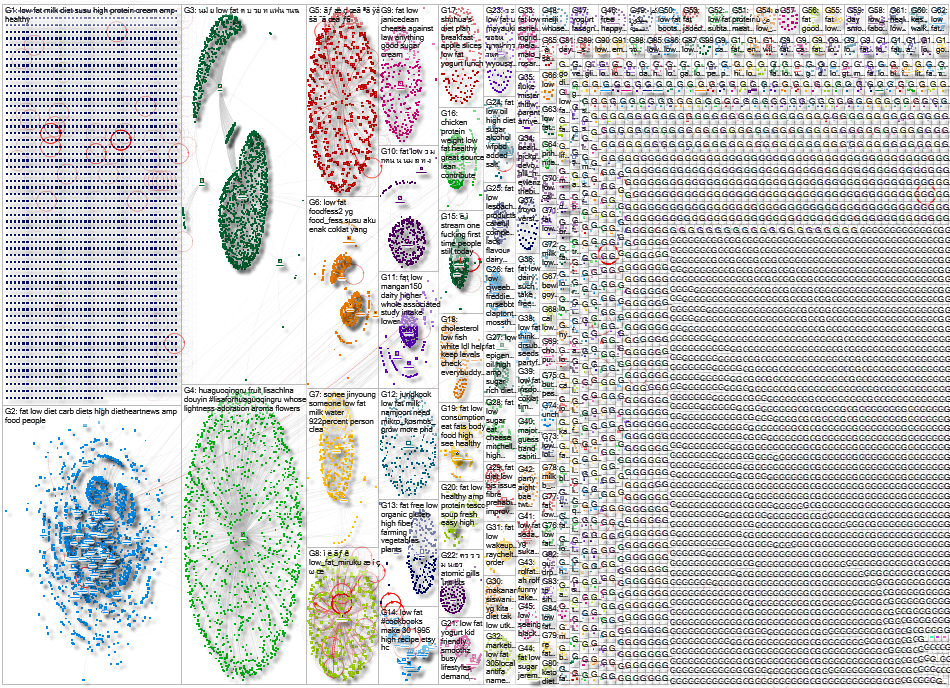 | **"soy free"** | 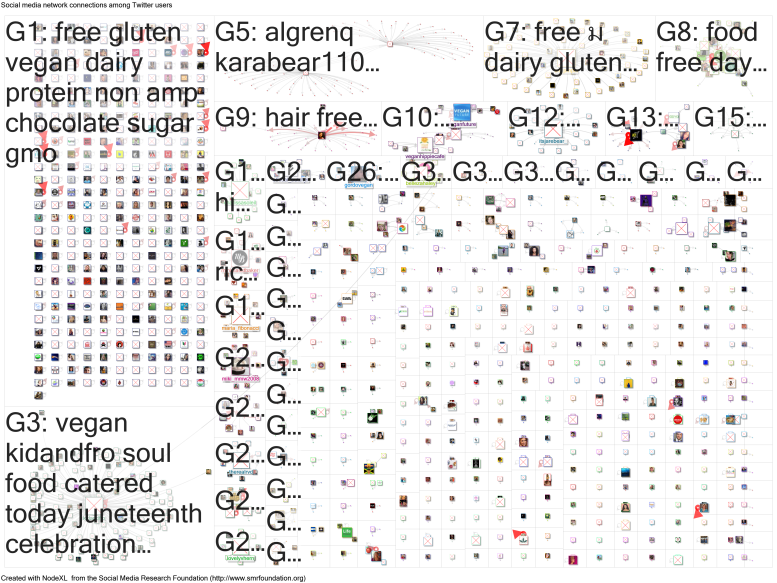 |
| **Graph Type** | *Community cluster network, where brand cluster is dominant and all other clusters are broadcast.* | **Graph Type** | *Community cluster network, where brand cluster is dominant and all other clusters are broadcast* |
| **“dairy free”** | 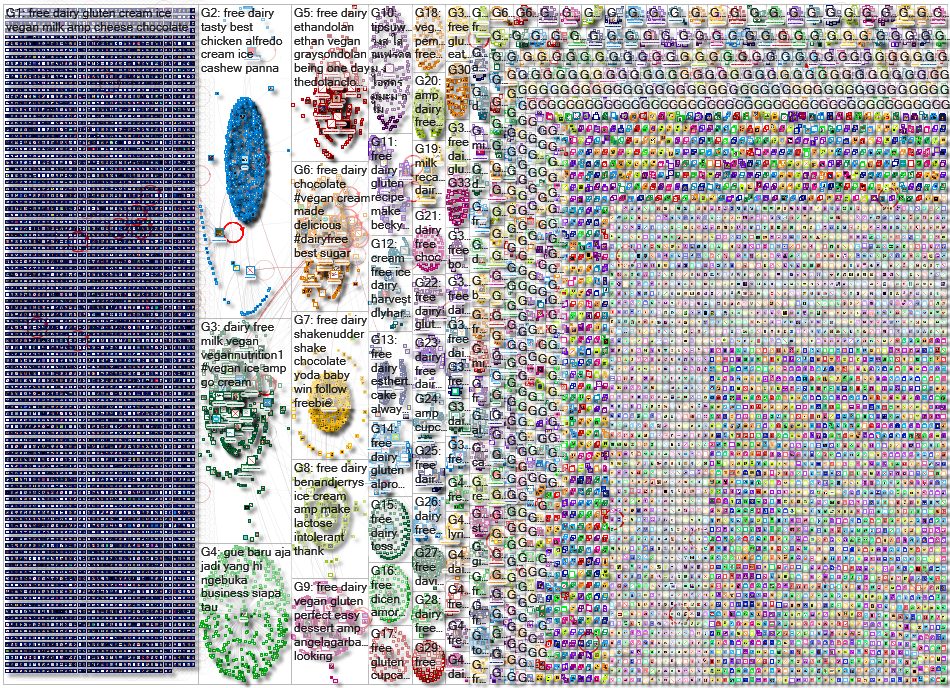 | **Intermittent Fasting** | 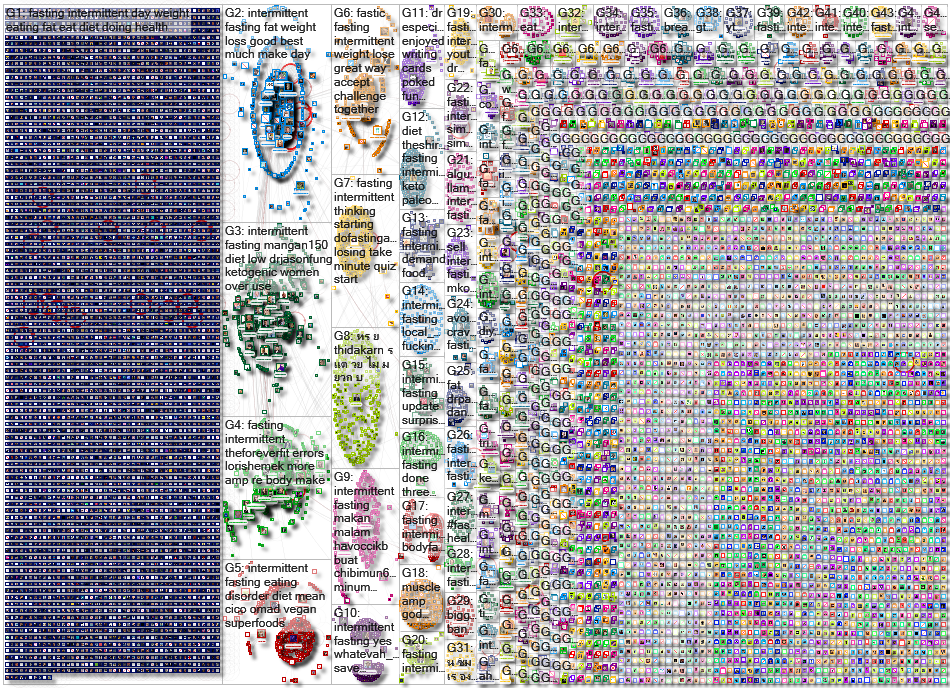 |
| **Graph Type** | *Community cluster network, where brand cluster is dominant, there is possible evidence of community interaction in group 3 and all other clusters are broadcast.* | **Graph Type** | *Community cluster network, where brand cluster is dominant, there is possible evidence of community interaction in group 3 and 4, and all other clusters are broadcast.* |
| **lchf** | 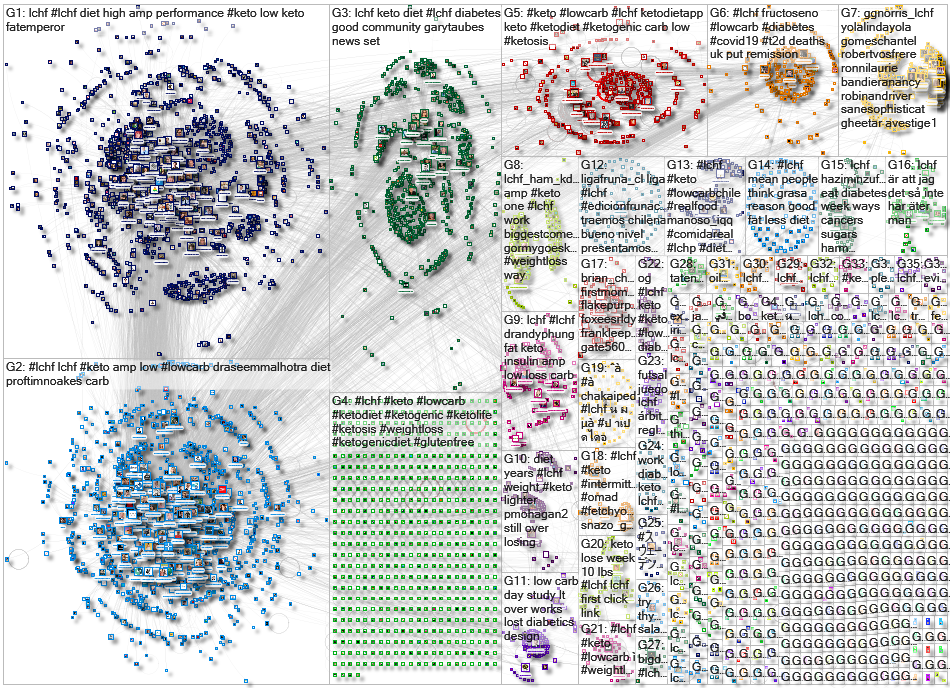 | **“low carb”** | 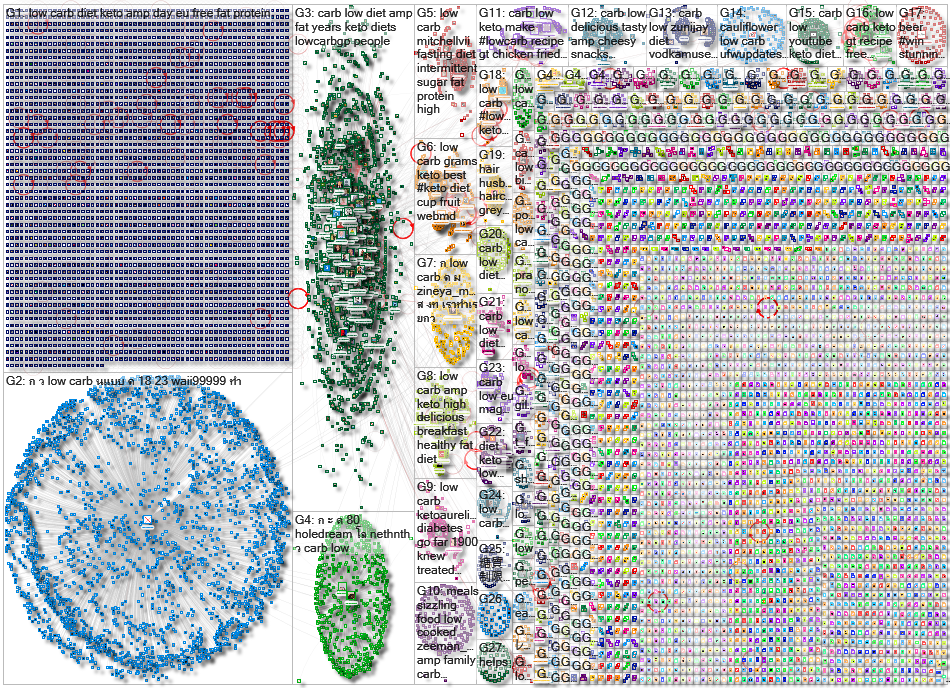 |
| **Graph Type** | *Broadcast network, where broadcast is dominant and a brand cluster is in group four.* | **Graph Type** | *Community cluster network, where brand cluster is dominant and all remaining clusters are broadcast.* |
| **“South beach diet”** | 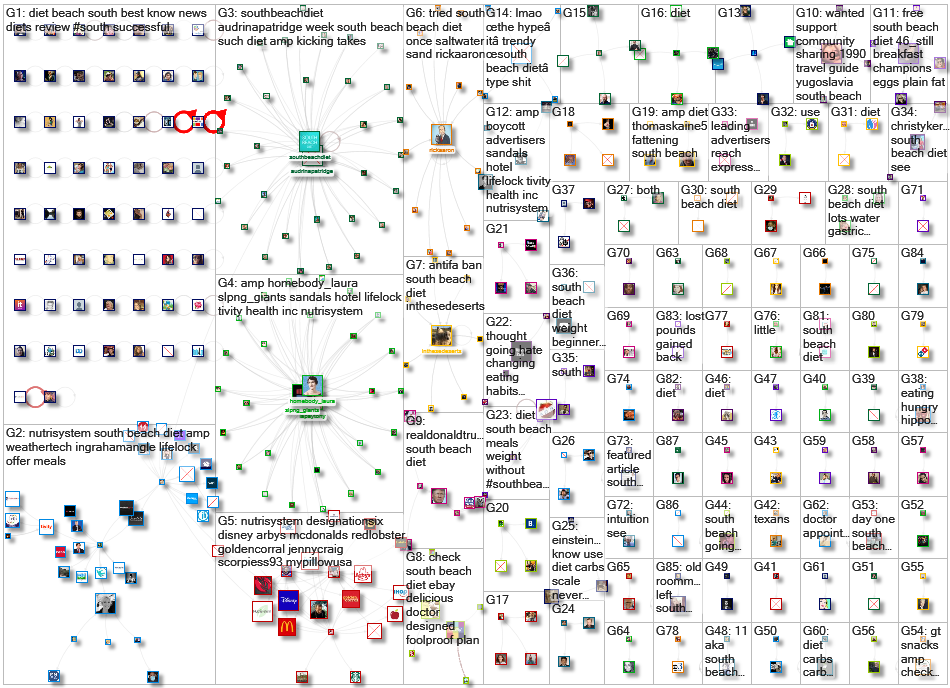 | **vegan** | 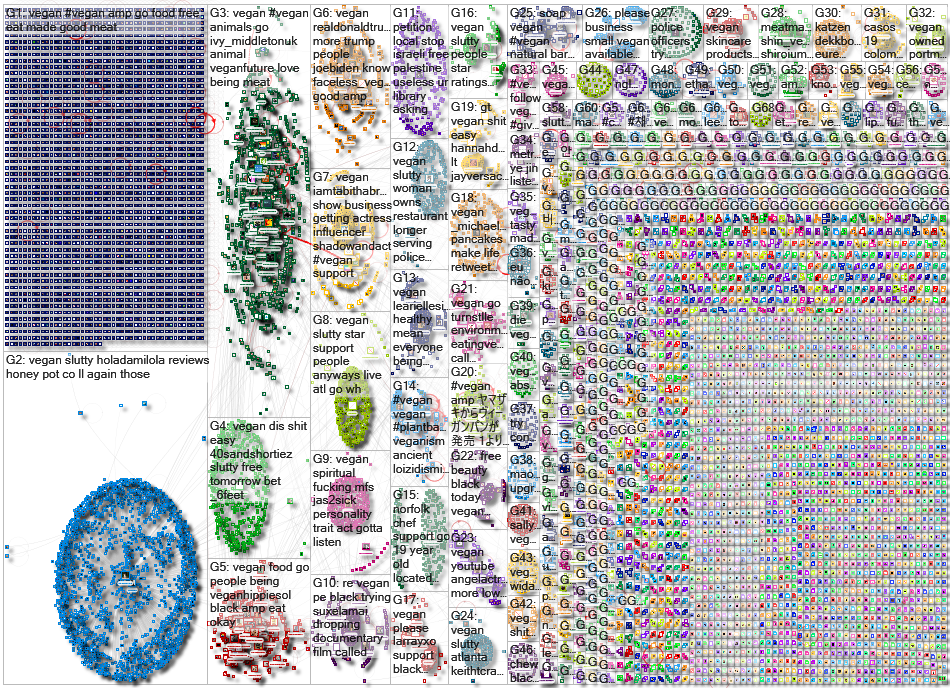 |
| **Graph Type** | *A smaller community cluster network where the brand cluster network is dominant, and all remaining clusters are broadcast.* | **Graph Type** | *Community cluster network, where brand cluster is dominant follow by a broadcast, possible evidence of community interaction in Group 3 and all remaining clusters are broadcast* |

Key:

Figure structures [56].


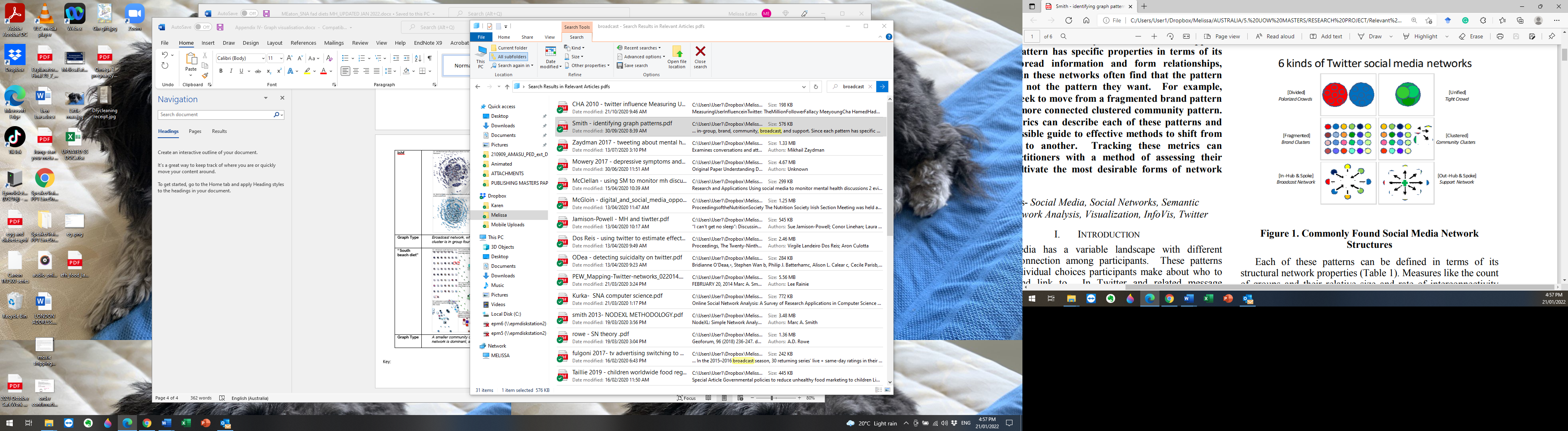

Supplement: Multimedia Appendix 4 [file infodemiology_v3i1e38245_app4.docx]
