# Supplementary material for: Characterizing the Discourse of Popular Diets to Describe Information Dispersal and Identify Leading Voices, Interaction, and Themes of Mental Health: Social Network Analysis
Source: JMIR Infodemiology. 2023 May 5;3:e38245. doi: 10.2196/38245 (PMC10199384; doi:10.2196/38245)
Supplement: Multimedia Appendix 5 [file infodemiology_v3i1e38245_app5.docx]

**Appendix V – Summary table**

**Table 1**. Influential users ranked by betweenness centrality and the mental health word frequency for depression and anxiety and eating disorders within the 16 popular diet networks.

|  | **Top 10 users** | **Followers**^a^, n | **Endorses diet**  **(Y/N)**^b^ | **Top 10 active users after adjusting for out-degree**^c^ | **Followers**^a^, n | **Endorses diet (Y/N)**^b^ | **Mental health**  **word frequency:**  **depression and anxiety (%)** | | **Mental health**  **word frequency:**  **eating disorder (%)**^d^ |
| --- | --- | --- | --- | --- | --- | --- | --- | --- | --- |
| **Online Network** |  |  |  |  |  |  |  | |  |
| **Paleo** |  |  |  |  |  |  | 0.009 (0.9) | | 0.031 (3.1) |
|  | Twitter user (“wellness”) ***A*** | 5020 | Unclear | “ |  |  |  |  | |
|  | *Dr (MD), personal brand (paleo protocol)* | 30,385 | Y | “ |  |  |  |  | |
|  | Online business (paleo recipes/food) | 1767 | Y | “ |  |  |  |  | |
|  | Twitter user (“health” blogger) | 2123 | Unclear | “ |  |  |  |  | |
|  | Twitter user (keto/LCHF) | 481 | Unclear | “ |  |  |  |  | |
|  | *Dr (MD), personal brand (carnivore diet)* | 31,936 | N | “ |  |  |  |  | |
|  | Twitter user (low-carb blogger) | 566 | Unclear | “ |  |  |  |  | |
|  | Video-sharing platform ***D*** | 72,272,825 | N | X |  |  |  |  | |
|  | *Dr (PhD, public health nutrition), personal brand (blog, media, and speaker)* | 58,635 | Unclear | “ |  |  |  |  | |
|  | Online business (keto challenge) | 87 | N | “ |  |  |  |  | |
|  |  |  |  | Brand, phone app ***G*** | 26,772 | Y |  |  | |
| **raw food** |  |  |  |  |  |  | 0.008 (0.8) | 0.005 (0.5) | |
|  | Government initiative (Nigeria) | 21,970 | N | “ |  |  |  |  | |
|  | Community initiative (Nigeria) | 82,301 | N | “ |  |  |  |  | |
|  | Twitter user (fan account) | 5521 | N | “ |  |  |  |  | |
|  | Twitter user (fan account) | 812 | N | “ |  |  |  |  | |
|  | Twitter user (fan account) | 2185 | N | “ |  |  |  |  | |
|  | Twitter user (fan account) | 11,705 | N | “ |  |  |  |  | |
|  | Twitter user (fan account) | 1624 | N | “ |  |  |  |  | |
|  | Twitter user (fan account) | 1030 | N | “ |  |  |  |  | |
|  | Twitter user (fan account) | 1903 | N | “ |  |  |  |  | |
|  | Twitter user (fan account) | 2174 | N | “ |  |  |  |  | |
| **vegan** |  |  |  |  |  |  | 0.011 (1.1) | 0.003 (0.3) | |
|  | Twitter user | 3231 | N | “ |  |  |  |  | |
|  | Twitter user | 2506 | Y | “ |  |  |  |  | |
|  | Twitter user (vegan) | 2741 | Y | X |  |  |  |  | |
|  | Twitter user | 22,454 | N | “ |  |  |  |  | |
|  | Online business (vegan “hub”) | 27,322 | Y | “ |  |  |  |  | |
|  | Twitter user (vegan) | 20,205 | Y | “ |  |  |  |  | |
|  | Online business (psychic) | 5050 | N | “ |  |  |  |  | |
|  | Online business (porn) | 1564 | N | “ |  |  |  |  | |
|  | Twitter user | 1623 | N | “ |  |  |  |  | |
|  | Twitter user | 4285 | N | “ |  |  |  |  | |
|  |  |  |  | President ***F*** | 82,406,667 | N |  |  | |
| **sugar free** |  |  |  |  |  |  | 0.007 (0.7) | 0.006 (0.6) | |
|  | Twitter user (fan account) | 14,138 | N | “ |  |  |  |  | |
|  | Television personality (Top Chef host) | 701,614 | N | “ |  |  |  |  | |
|  | Twitter user (food account) | 5404 | N | “ |  |  |  |  | |
|  | Twitter user | 110 | N | “ |  |  |  |  | |
|  | Twitter user (food blogger ***B)*** | 7064 | N | “ |  |  |  |  | |
|  | Brand (drink) | 36,806 | N | “ |  |  |  |  | |
|  | Twitter user (sugar-free food blogger) | 92,616 | Y | “ |  |  |  |  | |
|  | Online marketing company | 1,918,873 | N | “ |  |  |  |  | |
|  | Twitter user (chef and writer) | 7603 | N | “ |  |  |  |  | |
|  | Twitter user | 2486 | N | “ |  |  |  |  | |
| **dairy free** |  |  |  |  |  |  | 0.007 (0.7) | 0.003 (0.3) | |
|  | Twitter user (food blog) | 1,298,590 | N | “ |  |  |  |  | |
|  | Twitter user (fan account) | 2440 | Y | “ |  |  |  |  | |
|  | Twitter user (“wellness” ***A***) | 5058 | Y | “ |  |  |  |  | |
|  | *Vegan nutritionist, online business (nutrition consulting)* | 13,131 | Y | “ |  |  |  |  | |
|  | Twitter user | 4 | N | “ |  |  |  |  | |
|  | Brand (premade milkshakes) | 20,067 | N | “ |  |  |  |  | |
|  | Twitter user | 177 | N | “ |  |  |  |  | |
|  | Twitter user (food blogger ***B***) | 7064 | N | “ |  |  |  |  | |
|  | Brand (ice-cream) | 451,114 | N | X |  |  |  |  | |
|  | Brand (plant-based food) | 62,812 | Y | “ |  |  |  |  | |
|  |  |  |  | Twitter user | 492 | Y |  |  | |
| **gluten free** |  |  |  |  |  |  | 0.006 (0.6) | 0.003 (0.3) | |
|  | Twitter user, personal brand (production and voice-over) | 19,918 | N | “ |  |  |  |  | |
|  | Twitter user | 110,338 | N | “ |  |  |  |  | |
|  | Twitter user | 54 | N | “ |  |  |  |  | |
|  | Twitter user | 108 | N | “ |  |  |  |  | |
|  | Twitter user | 493 | N | “ |  |  |  |  | |
|  | Politician (US house candidate) | 7,676,083 | N | “ |  |  |  |  | |
|  | Actor | 2,478,403 | N | X |  |  |  |  | |
|  | Twitter user | 51 | N | “ |  |  |  |  | |
|  | Twitter user | 537 | Y | “ |  |  |  |  | |
|  | Twitter user | 3936 | Unclear | “ |  |  |  |  | |
|  |  |  |  | Twitter user | 31,883 | Unclear |  |  | |
| **low carb** |  |  |  |  |  |  | 0.009 (0.9) | 0.017 (1.7) | |
|  | Twitter user | 12,689 | Y | “ |  |  |  |  | |
|  | Twitter user (keto) ***E*** | 4029 | Y | “ |  |  |  |  | |
|  | *Dr (General Practitioner)* | 49575 | Y | “ |  |  |  |  | |
|  | Twitter user, personal blog/brand (carnivore diet) | 64162 | Y | “ |  |  |  |  | |
|  | Twitter user (personal weight loss journey)  ***H*** | 310 | Y | “ |  |  |  |  | |
|  | Twitter user | 2452 | Y | “ |  |  |  |  | |
|  | Twitter user | 4303 | N | X |  |  |  |  | |
|  | Twitter user | 12 | Unclear | “ |  |  |  |  | |
|  | *Microbiologist, personal brand and business* (“science-based” *health and fitness)* ***C*** | 94,886 | Y | “ |  |  |  |  | |
|  | Television host (YourVoice America) | 586625 | Unclear | X |  |  |  |  | |
|  |  |  |  | Online marketing company | 14264 | N |  |  | |
|  |  |  |  | Twitter user (low carb) | 233 | Y |  |  | |
| **low fat** |  |  |  |  |  |  | 0.008 (0.8) | 0.012 (1.2) | |
|  | Twitter user (fan account) | 20,976 | N | “ |  |  |  |  | |
|  | *Microbiologist, personal brand and business* (“science-based” *health and fitness)* ***C*** | 95,662 | N | “ |  |  |  |  | |
|  | Twitter user | 17,790 | N | “ |  |  |  |  | |
|  | Twitter user (fan account) | 13,373 | N | “ |  |  |  |  | |
|  | Television host (senior meteorologist at Fox News and author) | 176,892 | N | “ |  |  |  |  | |
|  | Twitter user | 190 | N | “ |  |  |  |  | |
|  | *Science journalist, adjunct professor (saturated fat advocate)* | 90,819 | N | “ |  |  |  |  | |
|  | Video sharing platform ***D*** | 72,205,680 | N | X |  |  |  |  | |
|  | Non-profit initiative (India) | 76 | N | “ |  |  |  |  | |
|  | Twitter user | 5943 | N | “ |  |  |  |  | |
|  |  |  |  | Twitter user | 3499 | N |  |  | |
| **zone diet** |  |  |  |  |  |  | 0.029 (2.9) | 0.034 (3.4) | |
|  | Twitter user | 133 | Y | “ |  |  |  |  | |
|  | Online business (blue zone diet) | 38,387 | N | “ |  |  |  |  | |
|  | Brand (lifestyle magazine) | 198,486 | N | “ |  |  |  |  | |
|  | Unknown (no description, account suspended) | 106 | Unclear | “ |  |  |  |  | |
|  | *Nutritionist* | 5433 | Y | “ |  |  |  |  | |
|  | Twitter user (science librarian) | 766 | N | “ |  |  |  |  | |
|  | Twitter user | 11,153 | Unclear | “ |  |  |  |  | |
|  | Unknown (no description, account suspended) | 100 | Unclear | “ |  |  |  |  | |
|  | Unknown (no description, account suspended) | 240 | Unclear | X |  |  |  |  | |
|  | Twitter user | 427 | N | X |  |  |  |  | |
|  |  |  |  | Twitter user (English as a second language teacher) | 841 | N |  |  | |
|  |  |  |  | *Nutritionist* | 44,574 | N |  |  | |
| **atkins diet** |  |  |  |  |  |  | 0.02 (2.0) | 0.024 (2.4) | |
|  | *Dietitian (Malaysia)* | 2097 | N | “ |  |  |  |  | |
|  | Twitter user | 693 | Unclear | “ |  |  |  |  | |
|  | Twitter user | 25,207 | N | “ |  |  |  |  | |
|  | President ***F*** | 82,406,667 | N | X |  |  |  |  | |
|  | Twitter user (blog, podcast, and musician) | 3892 | N | “ |  |  |  |  | |
|  | Twitter user (Atkins for epilepsy) | 12,285 | N | “ |  |  |  |  | |
|  | Twitter user (dog account) | 130,179 | Unclear | “ |  |  |  |  | |
|  | Twitter user | 25 | N | “ |  |  |  |  | |
|  | Television host (Reverend, Chancellor) | 346,063 | N | X |  |  |  |  | |
|  | Twitter user | 230 | N | “ |  |  |  |  | |
|  |  |  |  | Twitter user | 1086 | N |  |  | |
|  |  |  |  | Twitter user | 381 | N |  |  | |
| **south beach diet** |  |  |  |  |  |  | 0.016 (1.6) | 0.011 (1.1) | |
|  | Brand, weight loss (south beach diet) | 18,797 | Y | “ |  |  |  |  | |
|  | Twitter user | 295 | N | “ |  |  |  |  | |
|  | Twitter user | 312 | N | “ |  |  |  |  | |
|  | Twitter user | 1491 | N | “ |  |  |  |  | |
|  | Brand (resort) | 248,462 | N | X |  |  |  |  | |
|  | Brand (automotive) | 4158 | N | X |  |  |  |  | |
|  | Twitter user | 1282 | N | “ |  |  |  |  | |
|  | Television host (fox news) | 3,553,239 | N | X |  |  |  |  | |
|  | Brand (health) | 1297 | Y | “ |  |  |  |  | |
|  | Brand (weight loss) | 17,536 | N | X |  |  |  |  | |
|  |  |  |  | Television personality (south beach diet ambassador) | 2,436,196 | Y |  |  | |
|  |  |  |  | Twitter user | 3623 | N |  |  | |
|  |  |  |  | Twitter user | 475 | N |  |  | |
|  |  |  |  | Twitter user | 47 | N |  |  | |
| **keto** |  |  |  |  |  |  | 0.013 (1.3) | 0.019 (1.9) | |
|  | Video sharing platform ***D*** | 72,272,825 | N | X |  |  |  |  | |
|  | Twitter user | 1171 | N | X |  |  |  |  | |
|  | Twitter user (keto/carnivore) | 137 | Y | “ |  |  |  |  | |
|  | Twitter user | 18 | Y | “ |  |  |  |  | |
|  | Twitter user (keto/low-carb/LCHF/fasting) | 5255 | Y | “ |  |  |  |  | |
|  | *Dr (MD), personal brand (books, products, events, and blog)* | 59,909 | Y | “ |  |  |  |  | |
|  | Twitter user | 57,490 | N | X |  |  |  |  | |
|  | Twitter user | 1394 | N | “ |  |  |  |  | |
|  | Twitter user, personal brand (fitness and health coaching) | 33,884 | Y | “ |  |  |  |  | |
|  | Twitter user | 44,554 | N | X |  |  |  |  | |
|  |  |  |  | Twitter user (keto) ***E*** | 4028 | Y |  |  | |
|  |  |  |  | Twitter user (social media influencer) | 1,337,729 | N |  |  | |
|  |  |  |  | Twitter user | 418 | N |  |  | |
|  |  |  |  | Twitter user (personal weight loss journey)***H*** | 310 | Y |  |  | |
| **intermittent fasting** |  |  |  |  |  |  | 0.016 (1.6) | 0.024 (2.4) | |
|  | Twitter user | 378 | Y | “ |  |  |  |  | |
|  | Brand (intermittent fasting phone app) | 673 | Y | “ |  |  |  |  | |
|  | Twitter user, personal brand (intermittent fasting and fitness coach) | 14,492 | Y | “ |  |  |  |  | |
|  | Brand (vitamin supplements) | 341 | N | “ |  |  |  |  | |
|  | Twitter user (anorexia) | 206 | Unclear | “ |  |  |  |  | |
|  | Brand (intermittent fasting phone app) | 17,368 | Y | “ |  |  |  |  | |
|  | Twitter user | 155 | Y | “ |  |  |  |  | |
|  | *Dr (MD, nephrologist), personal brand (diet programs, coaching, and books)* | 128,478 | Y | “ |  |  |  |  | |
|  | *Dr (MD), personal brand (weight loss programs and health coaching* | 35,702 | Y | “ |  |  |  |  | |
|  | *Microbiologist, personal brand and business* (“science-based” *health and fitness)* ***C*** | 95,178 | Y | “ |  |  |  |  | |
| **detox diet** |  |  |  |  |  |  | 0.013 (1.3) | 0.021 (2.1) | |
|  | Twitter user (fan account) | 36,140 | N | “ |  |  |  |  | |
|  | Twitter user (weight loss products) | 62 | Y | “ |  |  |  |  | |
|  | Online business (“healing heat” therapy) | 3287 | N | “ |  |  |  |  | |
|  | Brand (Japanese magazine) | 458,635 | N | “ |  |  |  |  | |
|  | Twitter user (blogger, candida nutrition) | 1610 | Y | “ |  |  |  |  | |
|  | Brand (raw cold pressed juice) | 208 | N | “ |  |  |  |  | |
|  | Twitter user, online business (household services) | 14,522 | N | “ |  |  |  |  | |
|  | Twitter user | 397 | Unclear | “ |  |  |  |  | |
|  | Band (kpop) | 23,837 | N | X |  |  |  |  | |
|  | Twitter user, blog (organic food and products) | 137,707 | Unclear | “ |  |  |  |  | |
|  |  |  |  | Brand (health food shop) | 5823 | N |  |  | |
| **LCHF** |  |  |  |  |  |  | 0.011 (1.1) | 0.018 (1.8) | |
|  | *Journalist, science and health, personal brand* | 93,879 | Y | “ |  |  |  |  | |
|  | *Biochemical engineer, personal brand (blog, podcast, and book)* | 58,447 | Y | “ |  |  |  |  | |
|  | *Orthopedic surgeon, personal brand (blog and book)* | 55,635 | Y | “ |  |  |  |  | |
|  | *Emeritus professor, former MD, personal brand (blog, books, media, and nutrition coaching)* | 144,736 | Y | “ |  |  |  |  | |
|  | Twitter user (LCHF) | 545 | Y | “ |  |  |  |  | |
|  | Twitter user (LCHF) | 1926 | Y | “ |  |  |  |  | |
|  | Brand (keto phone app) ***G*** | 26,754 | Y | “ |  |  |  |  | |
|  | *Cardiologist, visiting professor, personal brand (media and books)* | 67,997 | Y | “ |  |  |  |  | |
|  | *Dietitian, professorial fellow, scientist, LCHF research for endurance athletes* | 12,099 | N | “ |  |  |  |  | |
|  | Twitter user (LCHF) | 2402 | Y | “ |  |  |  |  | |
| **soy free** |  |  |  |  |  |  | 0.005 (0.5) | 0.005 (0.5) | |
|  | Twitter user (vegan chef) | 21,028 | Y | “ |  |  |  |  | |
|  | Twitter user | 5702 | N | “ |  |  |  |  | |
|  | Twitter user | 3712 | N | “ |  |  |  |  | |
|  | Twitter user | 23,701 | N | “ |  |  |  |  | |
|  | Twitter user (vegan) | 1233 | N | “ |  |  |  |  | |
|  | Twitter user (soy, dairy, yeast and egg allergy) | 216 | Unclear | “ |  |  |  |  | |
|  | Online business (hair growth oil) | 52 | N | “ |  |  |  |  | |
|  | Twitter user | 1011 | N | X |  |  |  |  | |
|  | Twitter user | 794 | N | X |  |  |  |  | |
|  | Twitter user | 4954 | N | X |  |  |  |  | |
|  |  |  |  | Twitter user (vegan) | 80 | Unclear |  |  | |
|  |  |  |  | Twitter user | 2675 | Unclear |  |  | |
|  |  |  |  | Brand (food) | 90 | Unclear |  |  | |

a Follower count at the time of data collection

b Endorses popular diet as indicated by tweets collected and profile bio (Y=in support, N=not in support, and unclear=was not made clear)

c Active users adjusted by out degree (>1.0)

d Eating disorder word frequency after adjusting for sensitivity

***A-H*** = indicate reoccurring users

X= indicates that the account was removed after adjusting for out degree

“ = indicates that the ‘active user’ was the same account after adjusting for out degree

*Italics* = A user with an indicated health and/or science degree, background or profession

LCHF = Low-carbohydrate, high-fat

User categories: Twitter user (*account identify*), online business (*business type*), video-sharing platform, government initiative, community initiative, non-profit initiative, Television personality/host (*related show/identity*), actor, brand (*brand type*), online marketing company, personal brand (*brand identity*), politician, science/health related occupation eg *Dr, Medical Doctor [MD], PhD*, and *dietitian,* band (*band name*), and unknown
