# Supplementary material for: Characterizing the Discourse of Popular Diets to Describe Information Dispersal and Identify Leading Voices, Interaction, and Themes of Mental Health: Social Network Analysis
Source: JMIR Infodemiology. 2023 May 5;3:e38245. doi: 10.2196/38245 (PMC10199384; doi:10.2196/38245)
Supplement: Multimedia Appendix 6 [file infodemiology_v3i1e38245_app6.docx]

**Appendix VI – Top 10 Hashtags**

Table 1. Top 10 hashtags for 16 popular diet networks

| Diet network most mentioned – #keto; 2nd most mentioned - #vegan  Most mentioned across networks: #weightloss  Equal 3rd most common across networks: #healthy; #health; #recipes; #recipe | | | |
| --- | --- | --- | --- |
| **Diet** | **Top Hashtags** | **Diet** | **Top Hashtags** |
| paleo | paleo keto lowcarb glutenfree diet healthy ketodiet vegan lchf weightloss | "raw food" | healthylifestyle vegan rawfood corona food tharntypess2firstws 上尾 テイクアウト ガンバルあげお health |
| vegan | vegan govegan plantbased animalrights jesuswins crueltyfree vegetarian veganfood organic health | "sugar free" | lemonib sugarfree keto recipes lowcarb recipe ketorecipes paleo dessert healthy |
| "dairy free" | vegan dairyfree glutenfree recipe food recipes palmoilfree dragonsden cooking delicious | "gluten free" | glutenfree recipe vegan food recipes cooking delicious cook voiceover fiverr |
| "low carb" | lowcarb keto recipe weightloss recipes diet ketogenic ketorecipes ketodiet lchf | "low fat" | lisaforhuaguoqingru cookbooks recipe healthy dieting cookbookstores food nutrition lowfat weightloss |
| "zone diet" | diet bluezones nutrition weightloss rapamycin fatloss food bluezone longevity lifestyle | "atkins diet" | diet atkins keto health weightloss nutrition ketogenic recipes healthylifestyle fitness |
| "south beach diet" | south southbeach the southbeachdiet dieting cookbooks cookbookstores diet southbeachdietambassador heartwork | keto | keto ketodiet lowcarb ketogenic ketogenicdiet weightloss ketorecipes diet lchf ketolife |
| "intermittent fasting" | intermittentfasting fasting weightloss health if diet fatloss keto healthy wellness | "detox diet" | scoups detox diet weightloss health smoothie lockdown healthy nutrition fitness |
| lchf | lchf keto lowcarb ketodiet ketogenic ketosis weightloss ketogenicdiet ketorecipes ketolife | "soy free" | vegan ad plantbased soyfree veganprotein 10x glutenfree veganbcaa amazon 10xathleticusa |
