# Supplementary material for: Characterizing the Discourse of Popular Diets to Describe Information Dispersal and Identify Leading Voices, Interaction, and Themes of Mental Health: Social Network Analysis
Source: JMIR Infodemiology. 2023 May 5;3:e38245. doi: 10.2196/38245 (PMC10199384; doi:10.2196/38245)
Supplement: Multimedia Appendix 7 [file infodemiology_v3i1e38245_app7.docx]

**Appendix VII – Text analysis for mental health word lists**

Table 1. Total words, word count, word frequency (salience), and percentage for depression and anxiety and eating disorder text analysis
